# Effect of intra-partum azithromycin on the development of the infant nasopharyngeal microbiota: A post hoc analysis of a double-blind randomized trial

**DOI:** 10.1016/j.ebiom.2022.104227

**Published:** 2022-08-19

**Authors:** Bakary Sanyang, Thushan I. de Silva, Abdoulie Kanteh, Abdoulie Bojang, Jarra Manneh, Wouter A.A. de Steenhuijsen Piters, Chikondi Peno, Debby Bogaert, Abdul Karim Sesay, Anna Roca

**Affiliations:** a Disease Control and Elimination, Medical Research Council Unit The Gambia at London School of Hygiene and Tropical Medicine, Banjul, Gambia; b The Genomics Core Lab, Medical Research Council Unit The Gambia at London School of Hygiene and Tropical Medicine, Banjul, Gambia; c The Florey Institute and Department of Infection, Immunity and Cardiovascular Disease, Medical School, University of Sheffield, Sheffield, UK; d Vaccines and Immunity, Medical Research Council Unit The Gambia at London School of Hygiene and Tropical Medicine, Banjul, Gambia; e London School of Hygiene and Tropical Medicine, London, UK; f Department of Paediatric Immunology and Infectious Diseases, Wilhelmina Children's Hospital/University Medical Center Utrecht, Utrecht, the Netherlands; g Centre for Infectious Disease Control, National Institute for Public Health and the Environment, Bilthoven, the Netherlands; h Centre for Inflammation Research, Queen's Medical Research Institute, University of Edinburgh, Edinburgh, UK

**Keywords:** Azithromycin, Intrapartum, Infant, Nasopharyngeal microbiota, West Africa

## Abstract

**Background:**

Sepsis is a leading cause of neonatal death. Intrapartum azithromycin reduces neonatal nasopharyngeal carriage of potentially pathogenic bacteria, a prerequisite for sepsis. Early antibiotic exposure has been associated with microbiota perturbations with varying effects. This study aims to understand the effect of intrapartum azithromycin intervention on the developing nasopharyngeal microbiota of the child.

**Methods:**

Using 16S rRNA gene sequencing, we analysed the microbiota of 343 nasopharyngeal samples collected from birth to 12 months from 109 healthy infants selected from a double-blind randomized placebo-controlled clinical trial conducted in the Gambia (PregnAnZI-1). In the trial, 829 women were given 2g oral azithromycin or placebo (1:1) during labour with the objective of reducing bacterial carriage in mother and child during the neonatal period. The post-hoc analysis presented here assessed the effect of the intervention on the child nasopharyngeal microbiota development.

**Findings:**

55 children were from mothers given azithromycin and 54 from mothers given placebo. Comparing arms, we found an increase in alpha-diversity at day-6 (p = 0·018), and a significant effect on overall microbiota composition at days 6 and 28 (R^2^ = 4.4%, q = 0·007 and R^2^ = 2.3%, q = 0·018 respectively). At genus level, we found lower representation of *Staphylococcus* at day-6 (q = 0·0303) and higher representation of *Moraxella* at 12 months (q = 0·0443). Unsupervised clustering of samples by microbial community similarity showed different community dynamics between the intervention and placebo arms during the neonatal period.

**Interpretation:**

These results indicate that intrapartum azithromycin caused short-term alterations in the nasopharyngeal microbiota with modest overall effect at 12 months of age. Further exploration of the effects of these variations on microbiome function will give more insight on the potential risks and benefits, for the child, associated with this intervention.

**Funding:**

This work was jointly funded by the Medical Research Council (UK) (MC_EX_MR/J010391/1/MRC), Bill & Melinda Gates Foundation (OPP1196513), and MRCG@LSHTM Doctoral Training Program.

**Research in context**

**Evidence before this study**

We searched PubMed using the terms “intrapartum azithromycin” AND “microbio*” AND “neonat*” which did not produce any result. There are four ongoing trials taking place in Africa and Southern Asia with an overall planned recruitment of 100,000 women in labour (see www.clinicaltrials.gov) aiming at evaluating the impact of intrapartum azithromycin on maternal and neonatal sepsis and mortality. Only one trial with this intervention has been completed (acronym PregnAnZI-1, conducted in The Gambia). In this proof-of-concept trial, we aimed at evaluating the impact of intra-partum azithromycin on maternal and neonatal bacterial colonization as a necessary step for sepsis. We showed a marked reduction of neonatal nasopharyngeal carriage of gram-positive bacteria (namely GBS, *S. aureus* and *S. pneumoniae*) and reduction of neonatal infection (non-severe). We reported genomic diversity of *S. aureus* strains (the main cause of neonatal sepsis in the region) both in mothers and newborns. So far, there are no direct measures of the effects of this intervention on the overall host bacterial community. If the ongoing trials of prophylactic intrapartum azithromycin show a public health impact, information on the impact of the intervention on the neonatal microbiome will be necessary to consider the implementation of this intervention at wider scale.

**Added value of this study**

Our study provides evidence on the impact of intrapartum azithromycin on nasopharyngeal microbiome development in the child, comparing effects on diversity and trajectory of development between children whose mothers were given intrapartum azithromycin versus those who received placebo. This study also provides insight on the initial dynamics in the early naive neonatal nasopharyngeal microbiome and how azithromycin could modulate these changes. The trial was conducted in a setting with sociodemographic conditions typical of sub-Saharan Africa, thus representative of regions of high neonatal sepsis burden.

**Implications of all the available evidence**

Our evidence shows that a single oral dose (2g) of intrapartum azithromycin may cause short-term alterations in the nasopharyngeal microbiota but does not affect the trajectory of the development of this microbial niche. Given azithromycin's broad range of activity, further investigations are necessary to understand its effects on other microflora and in other niches such as the gut, as well as the functions of these complex communities.

Alt-text: Unlabelled box

## Introduction

The last few decades have witnessed an important reduction in the mortality of children aged under five years.^1^^,^^2^ The greatest reduction, however, occurred in children older than 1 month, while neonatal mortality (death during the first 28 days of life) was not significantly reduced.^2^ Neonatal deaths, estimated at approximately 2·5 million annually, now account for 47% of the world's under-five fatalities.^3^ Almost 80% of these deaths occur in low and middle income countries (LMIC) typically in Southern Asia and Sub-Saharan Africa (SSA), where rates of neonatal mortality are at least ten times higher than in high income countries.^3^ In order to achieve the sustainable development goal number 3 by 2030,^4^ neonatal mortality needs to decrease to less than 12 per 1000 live-births in all countries. Therefore, strategies targeted at this vulnerable age group are urgently needed in regions of the world with the highest mortality rates.

Severe bacterial infections, mainly sepsis, are a leading cause of neonatal deaths.^5^ Newborns may be infected through the birth canal during delivery and through close physical contact with the mother in the first days of life. Bacterial colonization in mothers is especially common in resource-limited settings including SSA.^6^ Infection may also be acquired from environmental sources, especially under poor hygiene conditions.^7^

Azithromycin is a broad spectrum macrolide that has antimicrobial activity against gram-positive and gram-negative bacteria,^8^ including rapidly growing pyogenic bacteria such as *Staphylococcus* and *Streptococcus*.^9^ A recently conducted double-blind placebo controlled clinical trial (PregnAnZi-I), has demonstrated that oral intrapartum azithromycin reduces carriage of potentially pathogenic bacteria in the nasopharynx of newborns,^5^ and reduces maternal and neonatal infections.^10^ This effect likely resulted from a combination of reduced density of maternal microbiota,^5^ and a high concentration of azithromycin secreted in breast milk for at least four weeks.^11^

A variety of factors during the perinatal period, including but not limited to antibiotic use, have been shown to influence microbiota development.^12^, ^13^, ^14^ The neonatal period represents an important window of initialization of nasopharyngeal microbiota development, and alterations during this period may affect the trajectory of this development causing perturbations such as an imbalance or maladaptation of colonizing species.^15^ Early colonization patterns influence bacterial succession patterns over time, and distinct bacterial profiles identifiable at six weeks of age have been shown to predict microbiome stability and frequency of respiratory infections within the first two years of life.^16^^,^^17^

With increasing concerns on the risks of microbiota alterations with the use of antibiotics early in life,^13^, ^14^, ^15^ further investigations are warranted to better understand the overall effects of a prophylactic intervention such as intrapartum azithromycin on the infant microbiota composition. Hereby, we aim to assess the impact of a single oral dose of intra-partum azithromycin (2g) on the infant nasopharyngeal microbiota in the PregnAnZI-1 trial.^18^ Using a 16S-rRNA-based sequencing approach, we seek to understand the effects on diversity and dynamicity of the microbiota from birth up to 12 months of age. This post-hoc study provides vital data to determine the safety of using intrapartum azithromycin for the control of neonatal sepsis in SSA.

## Methods

### Trial design

The PregnAnZI-1 study (ClinicalTrials.org NCT01800942) was a phase-III, double-blind, randomised, placebo-controlled trial in which 829 Gambian women in labour attending the Bundung Maternal and Child Hospital were randomized to receive a single dose of 2g of oral azithromycin or placebo (ratio 1:1).^18^ On average, the duration between administration of the intervention and delivery was 2 hours.^5^ Details of inclusion and exclusion criteria can be found elsewhere.^18^ After hospital discharge, women and babies were visited regularly for 2 months (daily visits for the first week, and weekly visits thereafter),^18^ and biological samples were collected during the first 4 weeks including nasopharyngeal swabs (NPS). If women or children were prescribed antibiotics during the follow-up period, sample collection was discontinued.^18^ For children born during the last 6 months of recruitment (Nov 2013 to Apr 2014; n=613), an additional home visit took place when the children turned 12 months. Approximately 75% of those children were sampled during this final post trial visit (n=461).^19^

### Selection of children for the microbiome analysis

**Figure 1:**
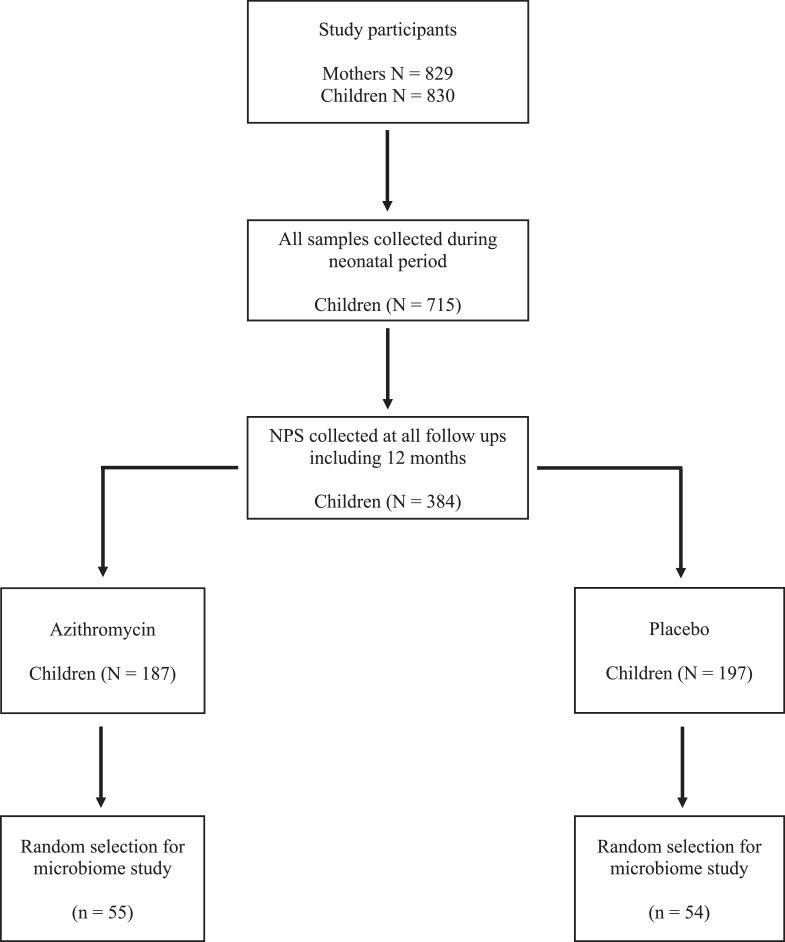
Selection of samples for microbiome study.

For this nested post-hoc study, we selected children who were sampled at all time-points, including the 12 months visit. Out of the 461 children sampled at the 12 months visit, 384 (83·3%) also had all NPS collected at the previous time points. We randomly selected 110 of these children (55 children from each trial arm). One child from the placebo arm was selected twice in error, making a total of 109 participants (55 azithromycin, 54 placebo). A summary of the study profile is shown in Figure 1. Overall, we included 436 samples for the microbiota analysis (4 time-points per selected participant): at birth (within 6 hours after birth), at day-6 (+/- 1 day), day-28 (+/- 3 days); and at 12 months (+/- 1 month) of age.

Sample size was calculated based on power to detect at least 10% difference in the abundance of the top 10 OTUs and 20% difference in the next 10 OTUs in the nasal microbiota. This was calculated using a tool developed by Mattiello *et al*,^20^ for calculating power and sample size for case-control microbiome studies. We estimated power using the anterior nares dataset from the human microbiome study embedded in the tool, which we anticipate has similar characteristics compared to our nasopharyngeal microbiota data. With this, a sample size of 40 per treatment group gave over 90% power to detect the hypothesized differences (please see supplementary Figure 1). We selected an excess of 15 samples in anticipation of dropouts during laboratory processing and data analysis.

### DNA extraction and quality control

DNA extraction was done using DNeasy® PowerLyzer® PowerSoil® Kit from Qiagen (Qiagen, Germany) following the manufacturer's protocol. 50 µl of homogenized NPS in 2 ml of STGG transport medium was used as input and beat beating at 2500 RPM for 45 seconds was applied. Blank extraction controls were included and taken through all downstream analyses. DNA was quantified using Qubit 3·0 Fluorometer (invitrogen/thermoscientific) and then concentrated using a vacuum concentrator. Total DNA concentrations of samples are summarised in Supplementary Figure 2.

### Library preparation and sequencing

16S libraries were made following Illumina's protocol for 16S-rRNA gene amplification and library preparation using V3-V4 primers as described elswhere.^21^ A mock community consisting of 12 bacterial species similar to those found in the nasopharynx was included. Samples that did not show bands on gel and had concentrations similar to the negative PCR controls were excluded. Sequencing was done on a MiSeq (Illumina Inc., San Diego, CA) using the paired-end V3 kit (600 cycle).

### Bioinformatics analysis

#### Quality filtering and denoising

Quality of raw sequences was assessed using FastQC,^22^ (version 0·11·8). Sequences were trimmed using trimmomatic (version 0·39).^23^ Due to a drop in quality in the first 15 cycles of one sequencing run, the first 20 bases of all sequences included in this analysis were trimmed. This was followed by a conditional trimming of the leading and trailing ends when base quality score fell below Q30 and Q25, respectively. Paired trimmed sequences were analysed using mothur,^24^ (version 1.44.0) following the miseqSOP.^25^ After denoising and chimera removal, sequences were classified using the Silva rRNA gene database (Silva version 132) by naive Bayesian classification using the Wang method,^26^ at a bootstrap confidence of 80%. Sequences classified as unknown were removed. The remaining sequences were binned into operational taxonomic units (OTUs) at a distance cut-off of 0·03. Rare OTUs with sequence counts <2 or present in <10% of the samples were excluded. Contaminant OTUs (63 in total) identified using the R Decontam package (Version 1·10·0) using both the frequency and prevalence modes were removed from the dataset. A total of 415 OTUs remained after all filtering.

#### Microbial community analysis

The dataset was rarefied by sub-sampling to 2000 sequences for each sample with 1000 iterations, before diversity estimation to avoid bias due to uneven sequencing depths. Alpha diversity was estimated by Shannon index. Overall microbiota composition (beta diversity) was calculated using Bray-Curtis dissimilarity index. Visualizations were done in R (version 4·2·0) and RStudio (version 1.4.1103).^27^ We went further to cluster samples into metacommunities based on community similarity by probabilistic modelling using Dirichlet's Multinomial Mixture Models (dmm).^28^ This was executed with the get.communitytype command in mothur which modelled the data into increasing number of community types using dmm. The best fit for each number of community types identified was determined by Laplace approximation.

### Statistical analysis

Alpha-diversity was compared between trial arms at each time-point by two-tailed independent t-test assuming equal variance. P-values were adjusted by Benjamini_Hochberg correction. Normality of alpha diversity measures per time-point was confirmed by Shapiro-Wilk test. Changes in alpha-diversity over time within each arm was assessed using linear mixed effects models, including maternal age, ethnicity, and sex of child as mixed effects. P-values for pairwise comparisons of time-points were adjusted by Dunnett's test. Overall community composition was compared between trial arms per time-point by permutational multivariate analysis of variance (PERMAMOVA), restricting permutations within trial arm. Variance in overall microbiota composition by age was also estimated for either arm of the trial by PERMANOVA, restricting permutations withing subjects. Distribution of metacommunities, identified by unsupervised clustering, between trial arms was compared per time-point using Fisher's exact test. We assessed differential abundance of taxa between the trial arms at each time-point using the Zero-inflated Gaussian mixture model in the metagenomeSeq package in R. P-values of differential abundance were corrected for false discovery (FDR). In each group, OTUs representing <0·1% of the total sequences were not considered in differential abundance comparison to reduce background noise.

### Ethical approval

This study was approved by the MRC-Gambia Government Joint Ethics Committee.

### Role of funding source

The funders had no role in the design and implementation of the study, or analysis and publication of the results.

## Results

### Description of study population

Baseline characteristics of the selected 109 participants are shown in Table 1. Both groups were comparable at baseline.

**Table 1:** Summary of baseline characteristics of the trial arms.

Ethnicity, *n* (%)	Azithromycin (N=55)	Placebo (N=54)	p-value
Mandinka	22 (40.0)	25 (46.3)	0.417
Wollof	9 (16.4)	7 (13.0)	
Sarahule	2 (3.6)	0 (0)	
Jola	10 (18.18)	6 (11.11)	
Fula	6 (10.91)	11 (20.4)	
Others	6 (10.91)	5 (9.3)	
Maternal age (yrs), mean (SD)	27·0 (5·3)	25·3 (4·7)	0.101
Birth weight (Kg), mean (SD)	3·2 (0·38)	3·2 (0·32)	0·915
Sex, female (%)	32 (58·2)	25 (46·3)	0.294
Delivery season, wet (%)	37 (67·3)	35 (64·8)	0.945
Exclusive BF in first 6 months (%)	39 (70.9)	37 (68.5)	0.759
Predominant BF in first 6 months (%)	16 (29.1)	17 (31.5)	
Sample collection time			
day6 (days), median (IQR)	6 (6 – 6)	6 (6 – 6)	
day28 (days), median (IQR)	28 (27 – 28)	28 (27 – 28)	
1 year (months), median (IQR)	13 (12.8 – 13.5)	13 (12.8 – 13.4)	

SD, standard deviation; yrs, years; Kg, kilograms; wet season, June – October; BF, breastfeeding Predominant breastfeeding means breastfeeding was the main form of nutrition while complementary foods were given.

### Study samples

Out of the 436 samples (220 azithromycin, 216 placebo) selected, 93 (57 azithromycin, 36 placebo) did not pass all quality control steps and were therefore excluded from further analysis (Supplementary Table 1). From the day-0 samples, 20.2% were dropped due to no PCR amplification determined by electrophoresis and majority were from the azithromycin arm (18 vs 4 from the azithromycin and placebo arm respectively, p = 0·002).

### Alpha-diversity

The median read count after quality control was 40,023 (range: 2,071 – 143,800) for the samples, and 411 (range: 207 – 720) for the negative controls including STGG and blank extraction controls. Rarefaction curves show up to approximately 200 OTUs per sample for both azithromycin and placebo arms, at similar sequencing depths (Supplementary Figure 3).

**Figure 2:**
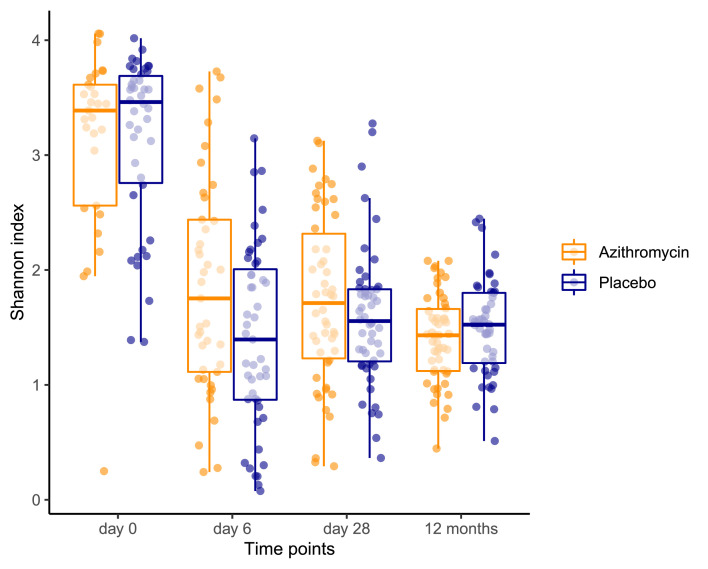
Box and whisker plot showing distribution of alpha-diversity scores between the trial arms at each time-point (day 0 p = 0.785, day 6 p = 0.031, day 28 p = 0.231, 12 months p = 0.234). The box shows the median and lower and upper quartiles (middle 50% of the scores) while the upper and lower whiskers show the upper and lower 25% scores respectively excluding outliers.

Alpha-diversity decreased between day-0 and day-6 (p < 0·001) for both azithromycin and placebo arms and was quite stable thereafter within each arm. We observed a higher diversity in the azithromycin arm at day-6 (p = 0·018) (Figure 2 and Supplementary Table 2). Diversity did not significantly differ at subsequent time-points.

### Beta-diversity

**Figure 3:**
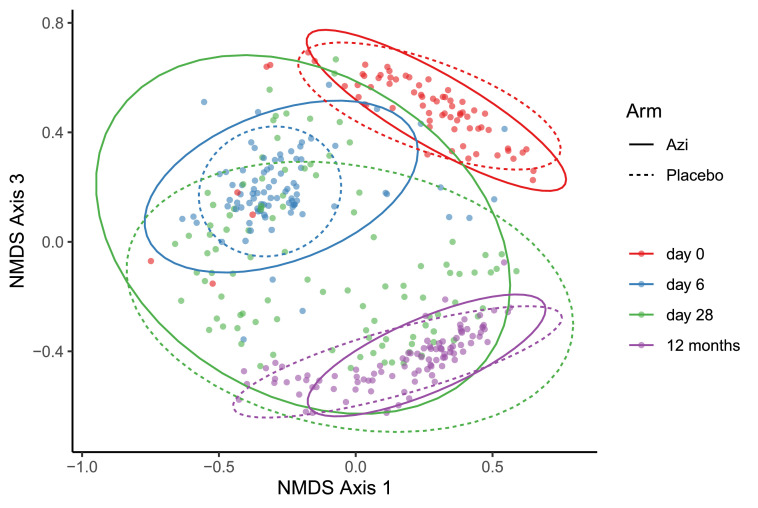
A non-metric multidimensional scaling (NMDS) plot showing bacterial community structure measured by Bray-Curtis dissimilarity index, compared by age in either trial arm and between trial arms at each age time-point using multivariate analysis.

We measured variance in overall microbiota composition (beta-diversity) with age and between the trial arms at each time point. Beta-diversity was highly driven by age in both trial arms as shown by PERMANOVA (Azithromycin R2 = 24·9%, q < 0·001; Placebo R2 = 26·9%, q = 0·007) (Figure 3). Beta-diversity also differed significantly between the trial arms at day-6 (R2 4·4%, q = 0.007) and day-28 (R2 2.3%, q = 0.018) but not at 12 months (R2 1·6%, q = 0·150) (Figure 3). Variance among individuals within each arm was only slightly higher for the azithromycin arm at day-6 (p = 0·059) but not at subsequent time-points (Supplementary Table 3).

### Community types by OTU clustering

**Figure 4:**
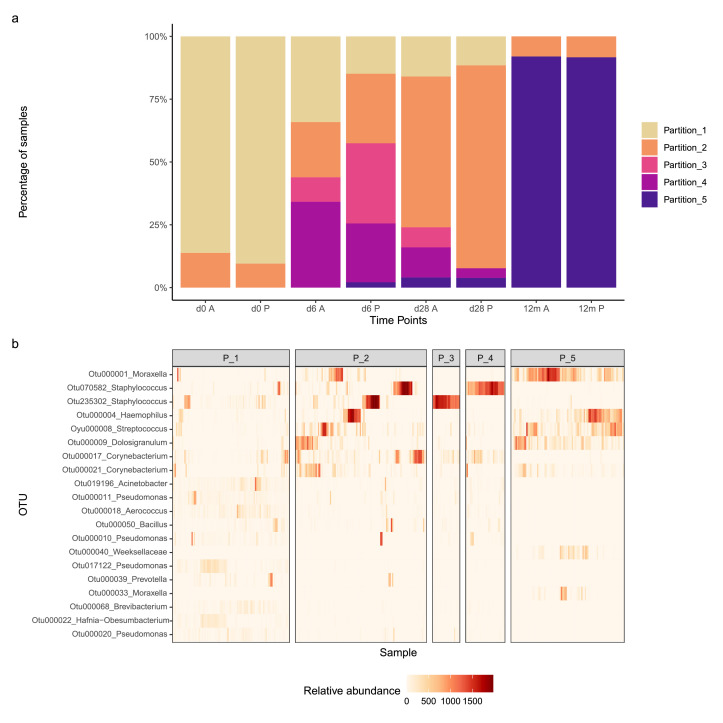
(a) Distribution of partitions generated by Dirichlet Multinomial Mixtures (dmm), clustering samples into community types (partitions) based on similarities of their community profiles. (b) A heatmap showing relative abundances of the top 20 OTUs in the dataset across the five partitions in “a”. A detailed list of main OTUs driving the individual partitions and their taxonomic annotations can be found in supplementary Table 4. d0 = day 0, d6 = day 6, d28 = day 28, 12m = 12 months, A = Azithromycin, P = Placebo, P_1 = Partition_1, P_2 = Partition_2, P_3 = Partition_3, P_4 = Partition_4, P_5 = Partition_5

Our clustering grouped samples into five metacommunities, referred here as partitions (Figure 4). Partition-1, dominated by recto-vaginal, gut, and environmental species, represented over 80% of the samples in both trial arms at day-0. Partition-2 was driven mostly by gram-positives including *Staphylococcus, Corynebacterium*, and *Streptococcus*, as well as some *Enterobacteriaceae* (gram-negative). This partition represented about 10% of the samples in both trial arms at day-0 and gradually increased during the first four weeks reaching 60% in the azithromycin arm, and 81% in the placebo arm by day-28. Partition-3, driven mainly by two *staphylococcus* OTUs, was highest at day-6 with lower representation in the azithromycin arm (10% azithromycin vs 32% placebo). At day-28, partition-3 was higher in the azithromycin arm (8% azithromycin vs 0% placebo). Partition-4 was driven by a *Staphylococcus* OTU which was seen at day-6 at slightly different proportions in the two arms (azithromycin 34% vs placebo 23%) and then declined in both arms at day-28. Partion-5, which dominated at 12 months (∼90% samples) in both arms, was driven by OTUs representing taxa that are common inhabitants of the infant nasopharynx. A detailed list of OTUs driving the different partitions can be found in supplementary Table 4. The data suggested different partition dynamics between trial arms which was confirmed by Fisher's exact test. There was an association of partitions frequencies with trial arm at days -6 and -28 (p = 0·024 and p = 0·069 respectively), but not at day-0 and 12 months (p = 0·708 and p = 1·0 respectively).

### Community profiles and differential abundance of taxa between trial arms over time

**Figure 5:**
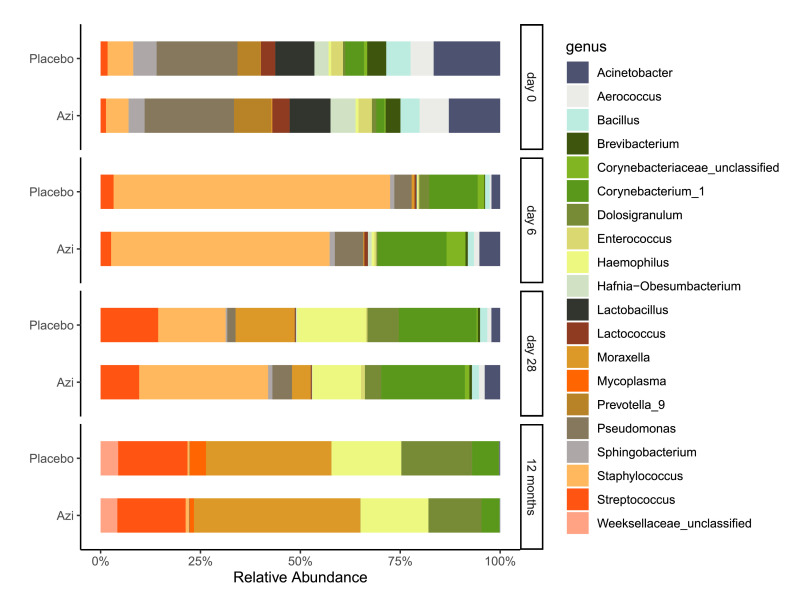
Genus profiles of trial arms grouped by time-point. The plot shows the mean relative abundance of the top 20 genera in either arm of the trial. The profiles show a dynamic trend from birth (day 0) to age 12 months, with some differences in taxa representation between arms.

**Figure 6:**
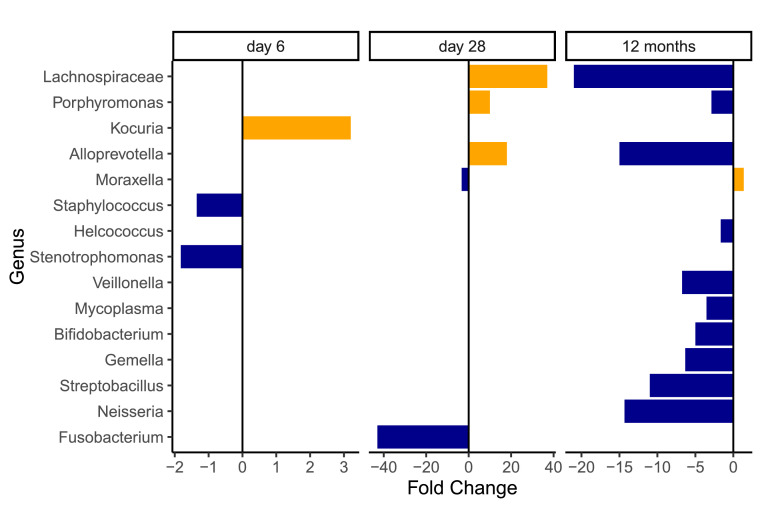
Genera that had significantly different representation between the trial arms grouped by time point. Horizontal axis shows fold change in abundance in the azithromycin arm compared to the placebo arm. Blue colour indicates reduction and yellow indicates increase. The magnitude of increase or decrease is indicated by the length and direction of the bar. Details on relative abundance of these genera in the trial arms and statistical significance of differences are listed in table supplementary Table 5.

Overall, both trial arms had similar phylum profiles across the time-points with six leading phyla – the gram positive Firmicutes and Actinobacteria, and gram negative Proteobacteria, Bacteroidetes, Fusobacteria, and Tenericutes (Supplementary Figure 4). Genus profiles were also generally similar between trial arms and in line with our alpha-diversity analysis showed a highly mixed profile at day-0 (Figure 5). We observed differential taxa representation between the trial arms both at genus and phylum levels at the different time points. This information is shown in Figure 6 and supplementary Table 5 (genus) and supplementary Table 6 (phylum). Day-0 samples were not included in differential abundance analysis due to small sample size.

At day-0, microbial communities in both arms were dominated by gram-negatives with >55% represented by Proteobacteria including *Pseudomonas, Acinetobacter, Sphingobacterium, Brevibacterium,* and *Prevotella* (Figure 5 and supplementary Figure 4). *Moraxella* and *Haemophilus,* at this point, were relatively low in abundance. Gram-positives were mostly *Lactobacillus, Staphylococcus, Lactococcus, Bacillus* and *Enterococcus*.

At day-6, however, gram-positives represented most of the community with 77% in the azithromycin arm and 88% in the placebo arm. Gram-positives were mostly *Staphylococcus* representing 49% in the azithromycin arm and 66% in the placebo arm (q = 0·030), followed by *Corynebacterium* (not significantly different, q = 0·572) (Figure 5 and supplementary Figure 4). Other differentially abundant genera between the trial arms were generally of low abundance (Supplementary Table 5).

At day-28, gram-positives still represented most of the community – 68% in the azithromycin arm and 60% in the placebo arm. These were mainly *Staphylococcus, Corynebacterium, Streptococcus*, and *Dolosigranulum*. Though at a low abundance, *Lachnospiraceae* was the only gram-positive that had a higher representation in the azithromycin arm (q = 0·022). Proteobacteria represented 28% in the azithromycin arm and 38% in the placebo arm (q = 0·543). Under this phylum, only *Moraxella* was differentially represented between the trial arms (4% in the azithromycin arm versus 14% in the placebo arm, q = 0·013). Also lower in the azithromycin arm was *Fusobacterium* (q = 0·043) of phylum Fusobacteria. Bacteroidetes had a higher representation in the azithromycin arm, driven by high abundance of *Alloprevotella* (q = 0·016) and *Porphyromonas* (q = 0·049).

At 12 months, gram-negatives represented >50% of the profiles in both arms. Proteobacteria was the leading phyla, dominated by *Moraxella* (40% in azithromycin arm, 29% in placebo arm, q = 0·044) and *Haemophilus* (16% in each arm). Firmicutes was the leading gram-positive phylum, dominated by *Streptococcus* (16% in each arm) and *Dolosigranulum* (13% in azithromycin arm, 17% in placebo arm). In both arms *Staphylococcus* representation was less than 1% and *Corynebacterium* less than 7%. Several genera were less represented in the azithromycin arm including *Mycoplasma* (q = 0·001), *Neisseria* (p < 0·001), and *Helcococcus* (q = 0·018).

## Discussion

Early antibiotic exposure has shown varying effects on host microbiota with the effect of a single dose of intrapartum azithromycin still unknown. This post hoc analysis of a randomized controlled trial shows that a single dose of 2g oral intrapartum azithromycin induced modest alterations in the overall nasopharyngeal bacterial community of the child that lasted for at least 28 days. Moreover, we observed different dynamics up to 28 days of life, and differences in abundance of multiple genera across different taxa up to 12 months.

In line with previous work, both alpha-diversity and beta-diversity were highly influenced by age. The sharp decline in alpha-diversity between birth and one week later is in agreement with what was shown in a similar cohort elsewhere.^29^ This is due to changing exposure and adaptation of colonizing bacteria to site-specific anatomical and physiological properties, in our case the nasopharynx.^30^, ^31^, ^32^ Age was an important driver of overall microbiota composition as well, similar to what was reported in a comparable South East Asian cohort,^33^ and in a recently published study in Netherlands.^30^ However, we observed a less dramatic change between day -6 and -28 in the azithromycin arm, probably as a result of delayed and/or decreased colonisation. In agreement with previous results from the main trial, such modulation of bacterial colonisation during the neonatal period could reduce risk of infection during this period of high vulnerability.^5^^,^^10^

Host and environmental factors as well as competition among colonizers play an important role in modulating microbial assembly. The upper airway naturally has a harsh microenvironment with limited free carbohydrate and glucose as a form of nutritional immunity to limit bacterial density.^34^ Under these nutrient poor conditions, bacterial competition plays a key role in the development of the microbiome. For example, *Corynebacterium accolens,* a member of the genus *Corynebacterium*, is known to release oleic acid from human nasal and skin surface triacylglycerols that suppress *S. pneumoniae* but favour growth of *S. aureus*.^34^ Looking at the community profiles over time, the relative abundance of *Corynebacterium* and *Staphylococcus* increased during the neonatal period, and then decreased at 12 months when firmicutes were dominated by *Dolosigranulum,* and *Streptococcus*. A similar pattern was also reported in South East Asia.^33^ The role of the environment is key in respiratory microbiome development, especially in LMIC. Although there is little data from LMIC, a review of house dust microbiome and its potential effects on human health reported that house dust is rich in gram-positive bacteria (mostly Firmicutes and Actinobacteria), including *Staphylococcus, Bacillus, Lactococcus*, and *Corynebacterium*.^35^ Bacterial communities were strongly driven by congestion, activities of occupants, and ventilation methods among others. In the Gambia, like other LMIC, household congestion and poor ventilation are common, which may further increase bacterial exposure during the neonatal period.^36^ Also, a recent publication on gut microbiota development in Gambian children showed differences in patterns of microbiota assembly between high income and low income settings,^37^ giving further support to this argument.

The higher fraction of day-0 samples that did not yield PCR products of the target gene in the azithromycin arm, was likely a result of the impact of intrapartum azithromycin on bacterial density in the birth canal, which resulted in lower transmission to the newborn as the maternal flora is an important contributor to microbiota seeding in the newborn.^32^ Therefore, the insignificant difference in the bacterial community at day-0 may be an artifact due to selection bias of samples with highest bacterial density in both arms. On the other hand, environmental sources of bacteria may have also contributed to this initial seeding profile.

The higher alpha-diversity at day-6 in the azithromycin was a result of enhanced evenness driven by the lower relative abundance of *Staphylococcus*. This is in line with the microbiology data from the main trial, which showed the highest prevalence and strongest difference between the trial arms, of *S. aureus* carriage at day-6.^5^ Control of bacterial over-proliferation, as shown by a lower proportion of the *Staphylococcus*-predominant cluster (partition-3) in the azithromycin arm, may have a modulatory effect on competition among colonizing bacteria and enhance diversity. This also translated into higher heterogeneity of the bacterial community among individuals in the azithromycin arm with varying relative abundances of *Staphylococcus* – which could reflect varying intake of azithromycin through breast milk, as its overall concentration at day-6 was highest but also most variable among mothers.^11^ These results are important as *Staphylococci* are among the leading bacteria causing neonatal sepsis is SSA,^38^ and the first week of life is a critical period when the incidence and mortality of sepsis are highest.^39^

Another important genus that had lower abundance in the azithromycin arm at day-6 was *Stenotrophomonas*, a ubiquitous bacterium in the environment including hospitals. So far, only one (*S. maltophilia*) out of eight identified species of this genus is known to cause disease in humans. *S. maltophilia* is an emerging multidrug resistant gram-negative bacteria associated with nosocomial infections including pneumonia, bacteraemia and septicaemia.^40^ On the contrary, two genera, Kocuria and Veillonella, were increased in the azithromycin arm though their abundances were low. The former has been found in the anterior nares with more abundance in an African than in a Western population.^41^ The latter was found in the nasopharyngeal microbiome of healthy similar aged children in rural Venezuela and was associated with less risk of gastrointestinal infection.^42^ Overall, these may indicate an effect of enhanced evenness, allowing increased representation by less prevalent species. However, with limited controls, we cannot rule out the possibility of this being influenced by environmental artifacts, given the low relative abundances of these genera.

At day-28, azithromycin did not have a significant impact on alpha-diversity, likely due to decreasing exposure of the child to azithromycin through breast milk over time.^11^ However, overall community composition was still different between the trial arms and, yet, with higher interpersonal variation in the azithromycin arm. This is likely driven by delayed dynamics in microbial assembly, thus impacting community structure. However, study children in both arms of the trial followed an overall normal trajectory of nasopharyngeal microbiome development. There was an overall increased representation of *Moraxella* (although lower representation in the azithromycin arm)*,* as well as other common genera found in the infant nasopharynx including *Corynebacterium, Haemophilus, Streptococcus*, and *Dolosigranulum* compared to the previous time-points, in both arms, indicating microbiome evolution due to changing exposure and niche specialization. In a Netherlands cohort, nasopharyngeal profiles characterized by the presence of *Moraxella* and *Corynebacterium* or *Dolosigranulum* at six weeks of age were associated with a stable microbiome with low frequency of respiratory infections in the first two years of life.^16^ Profiles with high abundance of *Haemophilus* and *Streptococcus* at six weeks were associated with a less stable microbiome.^16^ Fusobacterium, lower in the azithromycin arm, is a known inhabitant of the nasopharynx but with a generally low representation. *Alloprevotella, Porphyromonas,* and the family *Lachnospiraceae* which were higher in the azithromycin arm, are known commensals of the oral cavity and the gut. Their roles in the nasopharynx are not well characterized, although they have been associated with caesarean birth.^17^ The overall representation of *Staphylococcus* was not different between arms, however, the trend of higher proportion of partition-3 cluster in the azithromycin arm was likely due to selection for resistant *Staphylococci* as reported previously in the same cohort.^19^

By 12 months, intrapartum azithromycin did not have an effect on alpha diversity and overall microbiota composition, and profiles of both arms of the trial resembled a normal infant microbiome.^16^^,^^32^^,^^33^ Moreover, the two communities showed homogeneous clusters with about 90% of individuals in both arms belonging to partition-5, represented by common infant nasopharyngeal bacteria.^32^ Notwithstanding, differential representation of some taxa was observed, and among them, only *Moraxella* had a higher representation in the azithromycin arm. *Moraxella* is a key commensal of the infant nasopharynx and has been, with the exception of *M. catarrhalis*, associated with a healthy nasopharyngeal microbiome.^32^ The lower representation of *Mycoplasma* and *Neisseria* in the azithromycin arm could have resulted from modulatory effects of azithromycin on the initial microbiome. *Mycoplasma* species are atypical bacteria that are known to colonize the upper respiratory tract and include some pathogenic species such as *M. pneumoniae*, associated with asthma onset and symptom exacerbation.^33^, ^34^, ^35^ In a cohort of infants in China, incidence of respiratory *M. pneumonia* infection was highest among children 9 to 12 months old, who represented >40% of the cases.^43^ Similarly, in an Australian infant cohort, *Neisseria* co-colonisation with *Streptococcus* in the nasopharynx was strongly associated with higher respiratory infection.^44^ Therefore, a lower relative abundance of these bacteria in the azithromycin arm could have potential beneficial effects such as reduced frequency of respiratory infections, which, may in turn influence growth via the gut-lung axis. Anthropometric data from the same trial has shown less risk of malnutrition in the azithromycin arm, though this data is limited.^45^ Further investigation is needed to shed more light on the effect of the intervention on growth.

This study has several limitations in addition to the ones mentioned above. First, we conducted a retrospective analysis on samples collected from the PregnAnZi-1 trial which was not designed for microbiome analysis. Therefore, appropriate field controls were not available to identify and ascertain levels of contamination from the field. To some degree this may influence community profile and diversity, particularly at day-0, when nasopharyngeal biomass is very low. We minimised this effect by filtering out potential contaminant sequences before community analysis using the Decontam package using data from our blank library, STGG controls, and library concentrations of the samples. Any residual impact after the steps above is expected to be similar in both trial arms, except at day-0 given the potential lower biomass in the azithromycin arm. As a consequence of different biomass, any residual effect may impact differently at different age, with lower effect with increasing age. Secondly, most of the children selected for this study were born in the wet season compared to the main trial.^10^ This is due to the design of the trial as only children born in the last few months of the study were included in the 12 months follow-up. The rest of the children were too old to meet the entry criteria, that was being 11 to 13 months of age. Thirdly, we could not determine the impact of the intervention on the microbiota between day-28 and 12 months as samples were not collected during this period. Fourthly, due to the inherent limitation of 16S-based data, our interpretation of the effects of this intervention on the microbiome is limited to the genus level characterization of the bacterial community. Whole genome sequencing would give an understanding on the effects at species/strain level as well as the functional level. Fifthly, individuals selected for this analysis may not represent the overall population under study, especially because only healthy babies where included and potential seasonality effects were not considered. And lastly, we could not link our results to risk of respiratory infections including pneumonia among study participants as there was no active case detection between day-28 and 12 months. However, overall under-nutrition was lower in the azithromycin arm at 12 months, and the number of hospitalizations was also lower in the azithromycin arm (7 versus 3).

Understanding microbiota development is essential for better understanding of disease risk in different populations, particularly, in LMIC where limited data on microbiota development exists. Oral intrapartum azithromycin altered the relative abundance of some bacterial taxa in the nasopharynx of the infant but resulted in neither loss of diversity nor loss of key taxa. Inhibition of overgrowth of important gram-positive and gram-negative bacteria in the first week of life, shows the potential of intrapartum azithromycin to reduce risk of neonatal sepsis during the period of highest risk. Thus, this intervention could have a significant impact on the burden of neonatal sepsis caused by respiratory bacteria, especially, in LMIC where exposure to pathogens in the neonatal period is particularly high. Finally, similar analyses of the effect of intrapartum azithromycin on other niches including the gut is necessary to understand the overall potential risks and benefits for the child associated with this intervention.

## Contributors

This study, ancillary to the PregnAnZI-1 trial, was conceived, and designed by AR supported by AKS and BS. Sample selection was carried out by AB. The laboratory work including DNA extraction, library preparation, and sequencing was carried out by BS with support from JM. All laboratory work were supervised by AKS. Primary bioinformatics analysis was carried out by BS supported by AK. 16S microbial community analysis was carried out by BS supported by, TdS, DB, de WAASP, and CP. BS and AR drafted the manuscript. TdS, DB, WAASP, AKS, AK, AB and CP contributed to editing the manuscript. All authors approved the last version of the manuscript.

## Data sharing statement

The data in this study has been collected following provision of informed consent under the prerequisite of strict participant confidentiality. Qualified researchers may request access to collected and generated data with the Gambia Government/MRC Joint Ethics Committee. The review process and release of data will be facilitated by MRC Unit The Gambia (http://www.mrc.gm/) through the Head of Governance at MRCG, Elizabet Batchilli (ebatchilli@mrc.gm) and the corresponding author Prof Anna Roca (aroca@mrc.gm). Access will not be unduly restricted.

## Declaration of interests

We declare no conflicting interests.

## Appendix Supplementary materials

Image, application 1

Image, application 2

Image, application 3
