# Supplementary material for: Effect of intra-partum azithromycin on the development of the infant nasopharyngeal microbiota: A post hoc analysis of a double-blind randomized trial
Source: eBioMedicine. 2022 Aug 19;83:104227. doi: 10.1016/j.ebiom.2022.104227 (PMC9420482; doi:10.1016/j.ebiom.2022.104227)
Supplement: Supplementary file 1 [file mmc1.pdf]

Supplementary data

Table of contents

Table 1 – Samples removed during library preparation or data analysis…………………………………………………………………………2

Table 2 – Alpha diversity between trial arms and between age time-points within each arm…………………………………………………3

Table 3 – Homogeneity of group dispersion between trial arms, and by age in each trial arm……………………………………………….4

Table 4 – OTUs that were main drivers of microbiota clusters (partitions)………………………………………………………………...5

Table 5 – Genera with different abundances between trial arms…………………………………………………………………………7

Table 6 – Phyla with different abundance between trial arms……………………………………………………………………………8

Figure 1 – Sample size and power plot……………………………………………………………………………………..............8

Figure 2 – Initial DNA concentrations of samples and controls…………………………………………………………………………9

Figure 3 – Rarefaction curves of samples grouped by trial arm……………………………………………………………………...10

Figure 4 – Phylum profile of the samples grouped by trial arm and stratified by time-point……………………………………………………11

Table 1. Distribution of samples from Azithromycin and Placebo arms that were filtered and those that passed filtering before community analysis.

Time point	Trial Arm	No PCR product n (%)	Excluded from analysis n (%)	Included in analysis n (%)
Day 0	Azithromycin (N=55)	18 (32.7%)	15 (27.3)	22 (40.0%)
	Placebo (N=54)	4 (7.4%)	17 (31.5%)	33 (61.1%)
Day 6	Azithromycin (N=55)	11 (20.0%)	3 (5.5%)	41 (74.5%)
	Placebo (N=54)	4 (7.4%)	3 (5.6%)	47 (87.0%)
Day 28	Azithromycin (N=55)	4 (7.3%)	1 (1.8%)	50 (90.9%)
	Placebo (N=54)	2 (3.7%)	0 (0%)	52 (96.3%)
12 months	Azithromycin (N=55)	5 (9.1%)	0 (0%)	50 (90.9%)
	Placebo (N=54)	4 (7.4%)	2 (3.7%)	48 (88.9%)
Total	Azithromycin (N=220)	38 (17.3%)	19 (8.6%)	163 (74.1%)
	Placebo (N=216)	14 (6.5%)	22 (10.2%)	180 (83.3)

"No PCR product" means sample did not show any amplicon of the expected band size by electrophoresis. Samples "excluded from analysis" were those removed before analysis due to too low or too high read counts. These were samples with read counts below 2000 or four times higher than the average number of reads per sample (~4000). Samples passed were those that passed all quality filtering steps before community analysis.

Table 2. (a) Alpha diversity compared between trial arms at each age time-point estimated by Shannon index. p-values adjusted by Benjamini_Hochberg correction. (b) alpha diversity between successive age time-points in the azithromycin arm. (c) alpha diversity between successive age time-points in the placebo arm. P-values for multiple pairwise comparisons were adjusted by dunnett's test.

(a)

		**Mean Shannon Index**	**Df**	**p-value**
**Day 0**	Azi	3.03	53	0·782
	Placebo	3.09		
**Day 6**	Azi	1.86	86	0·018
	Placebo	1.41		
**Day 28**	Azi	1.73	100	0.270
	Placebo	1.59		
**12 months**	Azi	1.41	96	0·251
	Placebo	1.50		

Df =degrees of freedom.

(b)

Comparisons_Azithromycin	Estimate	Standard error	Df	p-value
Day 0 vs Day 6	-1.25	0.178	133	<0.001
Day 6 vs Day 28	-0.137	0.154	124	0.683
Day 28 vs 12 months	-0.306	0.145	119	0.0972

Degrees of freedom method: Kenward-roger

(c)

Comparisons_Placebo	Estimate	Standard error	Df	p-value
Day 0 vs Day 6	-1.25	0.178	133	<0.001
Day 6 vs Day 28	0.137	0.154	124	0.683
Day 28 vs 12 months	0.0956	0.126	139	0.764

Degrees of freedom method: Kenward-roger

Table 3. Homogeneity of Bray-Curtis dissimilarity (a) between time-points in the Placebo arm, (b) between time-points in the Azithromycin arm, and (c) between arms at each age time-point, measured by betadisper.

(a)

Time-points	P-value
day 0_P – day 6_P	0.392
day 6_P – day 28_P	0.720
day 28_P – 12 months_P	0.850

(b)

Time-points	P-value
day 0_A – day 6_A	0.597
day 6_A – day 28_A	0.097
day 28_A – 12 months_A	<0.627

(c)

Arm	P-value
day 0_A – day 0_P	0.985
day 6_A – day 6_P	0.059
day 28_A – day 28_P	0.112
12 months_A – 12 months_P	0.110

A = Azithromycin, P = Placebo

Table 4. Main OTUs driving the community types by unsupervised clustering

Partition_1		Partition_2		Partition_3		Partition_4		Partition_5	
**OUT**	**Taxonomy**	**OUT**	**Taxonomy**	**OUT**	**Taxonomy**	**OUT**	**Taxonomy**	**OUT**	**Taxonomy**
Otu000010	Pseudomonas	Otu000002	Staphylococcus	Otu235302	Staphylococcus	Otu070582	Staphylococcus	Otu000001	Moraxella
Otu000011	Pseudomonas	Otu000017	Corynebacterium_1	Otu000003	Staphylococcus			Otu000004	Haemophilus
Otu000018	Aerococcus	Otu000028	Escherichia-Shigella					Otu000008	Streptococcus
Otu000019	Pluralibacter	Otu000050	Bacillus					Otu000009	Dolosigranulum
Otu000020	Pseudomonas	Otu000055	Salmonella					Otu000012	Haemophilus
Otu000022	Hafnia-Obesumbacterium	Otu000090	Streptococcus					Otu000021	Corynebacterium_1
Otu000023	Pseudomonas	Otu000104	Gemella					Otu000033	Moraxella
Otu000026	Burkholderiaceae	Otu000117	Sphingobacterium					Otu000040	Weeksellaceae
Otu000027	Stenotrophomonas	Otu000118	Streptococcus					Otu000042	Suttonella
Otu000031	Rahnella	Otu000126	Janibacter					Otu000056	Streptococcus
Otu000034	Enterococcus	Otu000130	Micrococcus					Otu000080	Mycoplasma
Otu000041	Acinetobacter	Otu000149	Cutibacterium					Otu000087	Moraxella
Otu000045	Acinetobacter	Otu000153	Veillonella					Otu000126	Janibacter
Otu000048	Lactococcus	Otu000179	Kocuria					Otu000175	Helcococcus
Otu000049	Sphingobacterium	Otu000240	Kocuria					Otu024802	Moraxella
Otu000051	Acinetobacter	Otu017269	Acinetobacter						
Otu000057	Brachybacterium	Otu019269	Acinetobacter						
Otu000060	Flavobacterium								
Otu000063	Lactococcus								
Otu000065	Lelliottia								
Otu000067	Burkholderiaceae								
Otu000068	Brevibacterium								
Otu000070	Gardnerella								
Otu000071	Enterobacteriaceae								
Otu000074	Stenotrophomonas								
Otu000076	Comamonas								
Otu000082	Aquabacterium								
Otu000083	Enterococcus								

Otu000085	Sphingobacterium								
Otu000094	Rhizobiaceae_unclassified								
Otu000098	Serratia								
Otu000099	Diaphorobacter								
Otu000100	Curvibacter								
Otu000101	Microbacterium								
Otu000108	Brevundimonas								
Otu000124	Sphingobacterium								
Otu000152	Lysinibacillus								
Otu000161	Paucibacter								
Otu000171	Rhodoferax								
Otu000198	Cellulosimicrobium								
Otu000227	Sphingopyxis								
Otu000234	Methylobacterium								
Otu000340	Sphingopyxis								
Otu017122	Pseudomonas								
Otu017527	Acinetobacter								
Otu019196	Acinetobacter								

Table 5. Genera with different relative abundance between trial arms per age time-point. Only those that are statistically different between arms and have abundance above 0.1% are included in the table.

Time-point	Genus	Abundance_Placebo (%)	Abundance_Azi (%)	Fold change	q-value
Day-6	Staphylococcus	66.27	49.08	-1.35	0.0303
	Stenotrophomonas	0.51	0.28	- 1.82	0.0235
	Veillonella	0.00	0.17	NA	0.0235
	Kocuria	0.05	0.16	3.20	0.0255
Day-28	Moraxella	13.69	4.15	-3.30	0.0129
	Porphyromonas	0.09	0.90	10.00	0.0491
	Lachnospiraceae_unclassified	0.02	0.75	37.50	0.0223
	Alloprevotella	0.02	0.36	18.00	0.0158
	Fusobacterium	0.43	0.01	-43.00	0.0428
12 months	Moraxella	29.29	40.06	1.37	0.0443
	Mycoplasma	3.78	1.07	-3.53	0.0012
	Helcococcus	1.76	1.06	-1.66	0.0181
	Porphyromonas	0.49	0.17	-2.88	0.0007
	Bifidobacterium	0.20	0.04	-5.00	<0.0001
	Veillonella	0.27	0.04	-6.75	0.0056
	Neisseria	0.43	0.03	-14.33	<0.0001
	Gemella	0.19	0.03	-6.33	<0.0001
	Alloprevotella	0.30	0.02	-15.00	<0.0001
	Lachnospiraceae_unclassified	0.42	0.02	-21.00	<0.0001
	Streptobacillus	0.11	0.01	-11.00	<0.0001

Table 6. Differential abundance of phyla between trial arms at each time-point. Numbers indicate relative abundance (%) in normalised dataset.

Time-point	Phylum	Abundance_Placebo (%)	Abundance_Azi (%)	Fold change	q-value
Day-6	Firmicutes	74.22	56.22	- 0.76	0.0055
Day-28	Bacteroidetes	0.95	2.90	3.05	0.0070
12 months	Tenericutes	3.78	1.07	- 0.28	0.0002

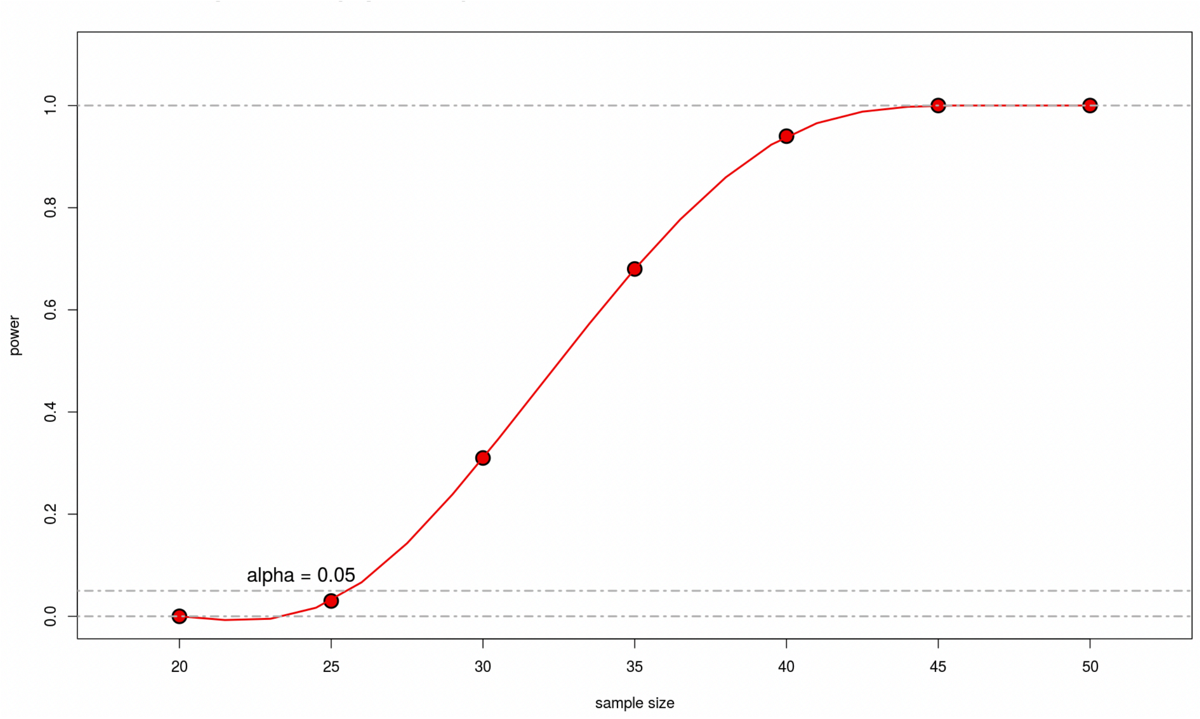

Figure 1. Sample size and power estimation for the study. Power was calculated by Monte Carlo simulations with 100 replications using the top 50 OTUs from the anterior nares dataset of the human microbiome study. The calculation was done for a case-control design using a sample size range of 20 – 50 per group, to detect a 10% difference in the top 10 OTUs and a 20% difference in the next 10 OTUs between the groups.

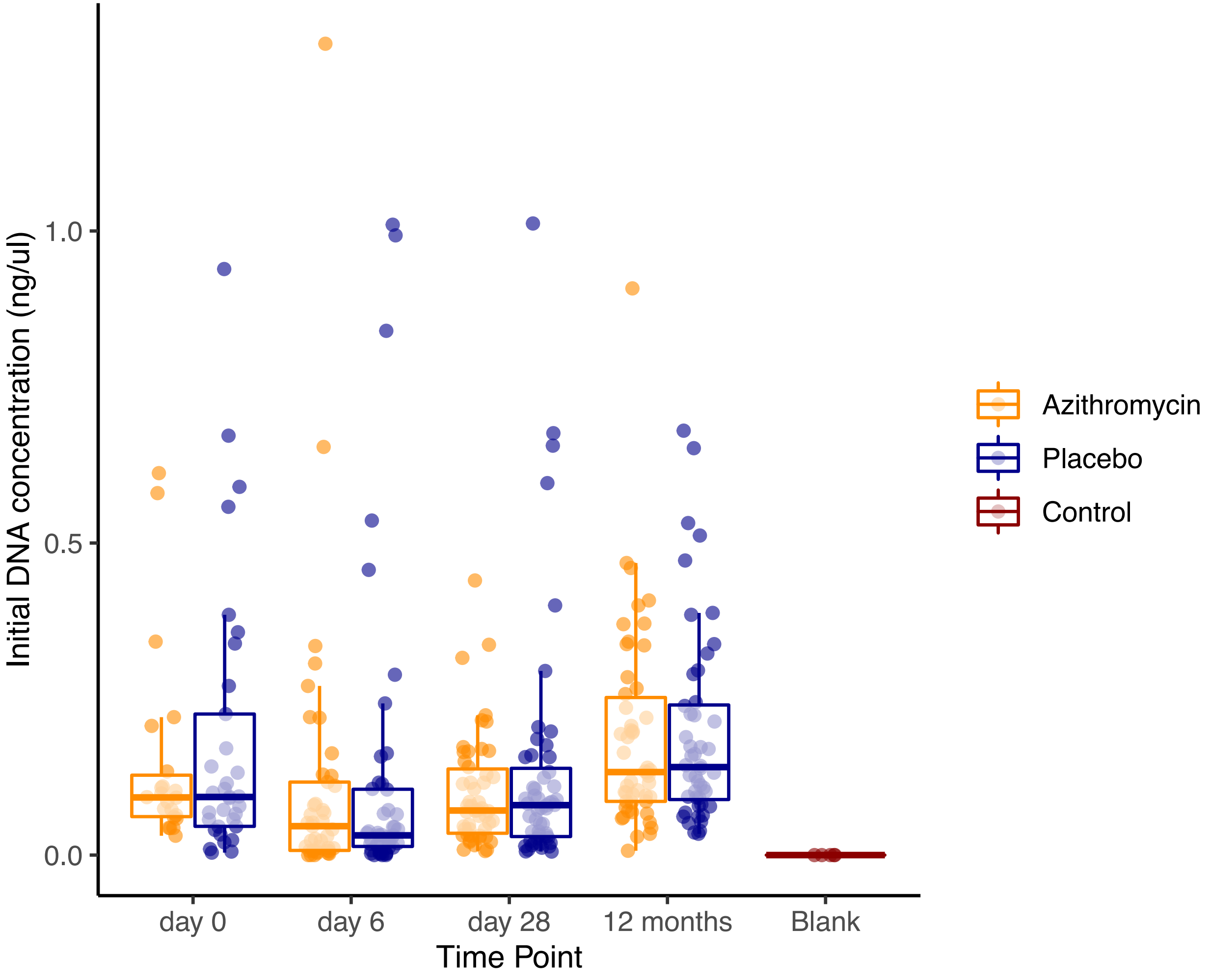

Figure 2. Initial DNA concentrations of samples included in the analysis grouped by trial arm and sample time point.

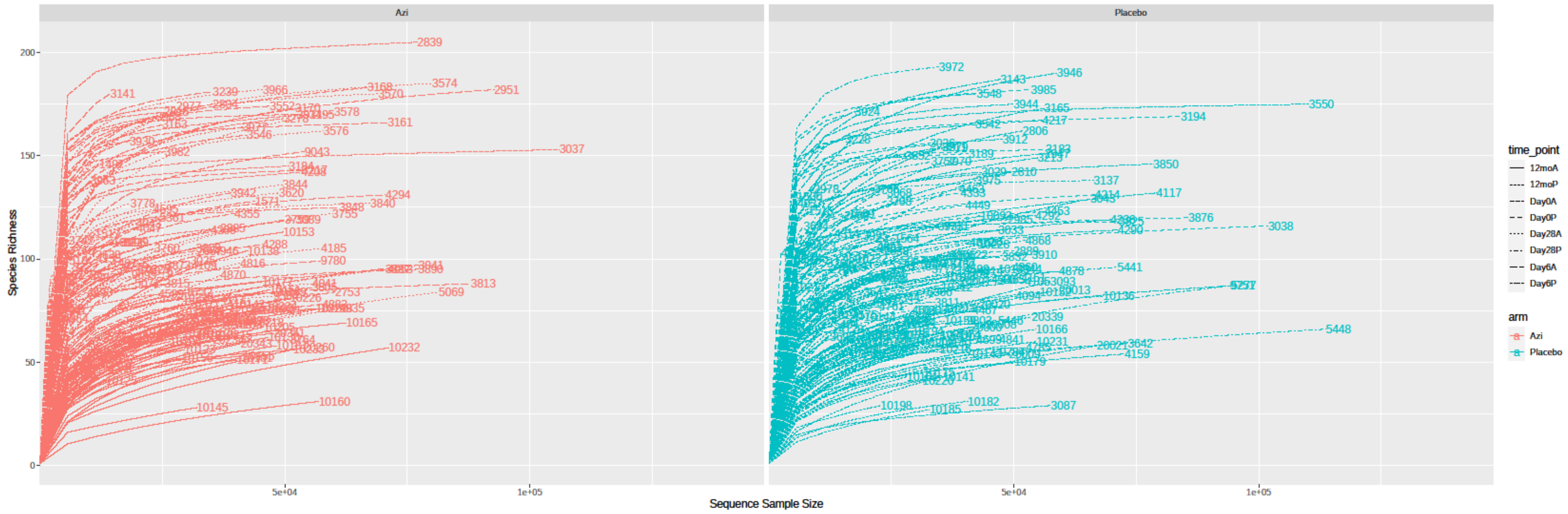

Figure 3. Rarefaction curves showing number of OTUs by sequencing depth for either trial arm. In both arms, OTUs reach asymptote at ~20000 reads.

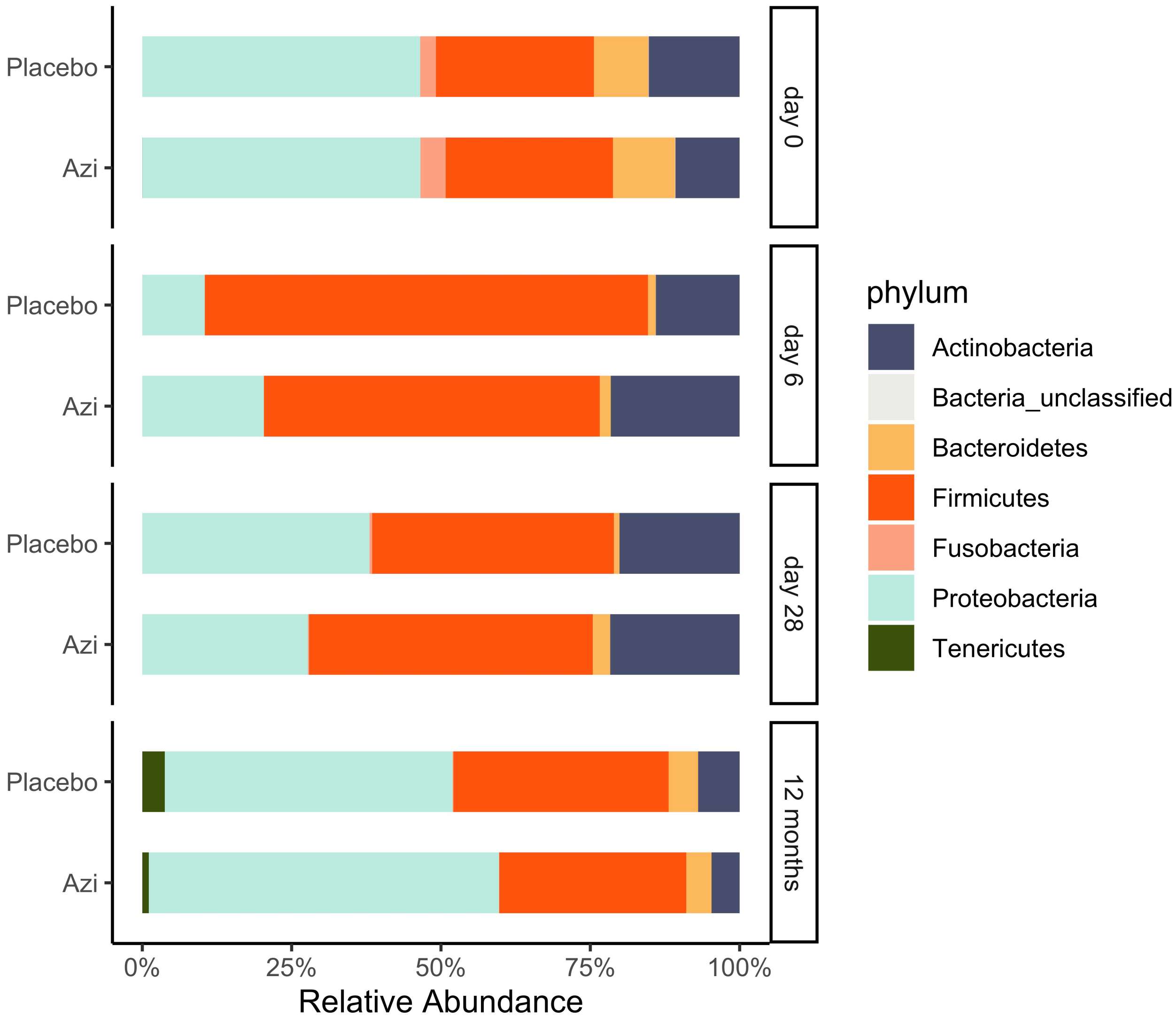

Figure 4. Phylum profile of the samples grouped by trial arm and stratified by time-point.
