# Supplementary material for: Effect of intra-partum azithromycin on the development of the infant nasopharyngeal microbiota: A post hoc analysis of a double-blind randomized trial
Source: eBioMedicine. 2022 Aug 19;83:104227. doi: 10.1016/j.ebiom.2022.104227 (PMC9420482; doi:10.1016/j.ebiom.2022.104227)
Supplement: Supplementary file 2 [file mmc2.pdf]

Roca et al. BMC Pregnancy and Childbirth (2015) 15:302
DOI 10.1186/s12884-015-0737-3

BMC Pregnancy & Childbirth

STUDY PROTOCOL Open Access

Prevention of bacterial infections in the newborn by pre-delivery administration of azithromycin: Study protocol of a randomized efficacy trial

Anna Roca[1,2*], Claire Oluwalana[1], Bully Camara[1], Abdoulie Bojang[1], Sarah Burr[2], Timothy M.E. Davis[3], Robin Bailey[2], Beate Kampmann[1], Jenny Mueller[1], Christian Bottomley[2] and Umberto D'Alessandro[1,2]

Abstract

Background: Neonatal deaths, estimated at approximately 4 million annually, now account for almost 40 % of global mortality in children aged under-five. Bacterial sepsis is a leading cause of neonatal mortality. Assuming the mother is the main source for bacterial transmission to newborns, the primary objective of the trial is to determine the impact of one oral dose of azithromycin, given to women in labour, on the newborn's bacterial carriage in the nasopharynx. Secondary objectives include the impact of the intervention on bacterial colonization in the baby and the mother during the first month of life.

Methods/design: This is a Phase III, double -blind, placebo controlled randomized clinical trial in which 830 women in labour were randomized to either a single dose of 2 g oral azithromycin or placebo (ratio 1:1). The trial included pregnant women in labour aged 18 to 45 years attending study health centres in the Western Gambia. A post-natal check of the mother and baby was conducted at the health centre by study clinicians before discharge and 8–10 days after delivery. Home follow up visits were conducted daily during the first week and then weekly until week 8 after delivery. Vaginal swabs and breast milk samples were collected from the mothers, and the pathogens *Streptococcus pneumoniae*, *Group B Streptococcus* (GBS) and *Staphylococcus aureus* were isolated from the study samples. For bacterial isolates, susceptibility pattern to azithromycin was determined using disk diffusion and E-test. Eye swabs were collected from newborns with eye discharge during the follow up period, and Chlamydial infection was assessed using molecular methods.

Discussion: This is a proof-of-concept study to assess the impact of antibiotic preventive treatment of women during labour on bacterial infections in the newborn. If the trial confirms this hypothesis, the next step will be to assess the impact of this intervention on neonatal sepsis. The proposed intervention should be easily implementable in developing countries.

Trial registration: ClinicalTrials.gov Identifier -NCT01800942- First received: February 26, 2013.

Keywords: Bacterial colonization, Neonatal sepsis, Azithromycin, Randomized clinical trial, Sub-Saharan Africa

* Correspondence: aroca@mrc.gm
[1] Medical Research Council Unit, Fajara, The Gambia
[2]London School of Hygiene and Tropical Medicine, London, United Kingdom
Full list of author information is available at the end of the article

BioMed Central

© 2015 Roca et al. **Open Access** This article is distributed under the terms of the Creative Commons Attribution 4.0 International License (http://creativecommons.org/licenses/by/4.0/), which permits unrestricted use, distribution, and reproduction in any medium, provided you give appropriate credit to the original author(s) and the source, provide a link to the Creative Commons license, and indicate if changes were made. The Creative Commons Public Domain Dedication waiver (http://creativecommons.org/publicdomain/zero/1.0/) applies to the data made available in this article, unless otherwise stated.

Background

The last decade witnessed a substantial reduction in the risk of under-five child mortality [1–3]. However, the greatest reduction occurred in children older than 1 month. Neonatal deaths, estimated at approximately 4 million annually, now account for almost 40 % of the world's deaths in children under-five [4] with approximately 75 % of these deaths occurring during the first week of life [5]. The highest rates of mortality in this age group occur in Sub-Saharan Africa (SSA).

Invasive bacterial disease, which mainly presents as sepsis, is estimated to cause one out of three deaths in this age group [5]. In the African continent, neonatal sepsis is caused by a wide range of bacterial pathogens [6] that differ between the early (first week of life) and late (days 8 to 28) neonatal period. During the early neonatal period, when most neonatal sepsis occur [7], gram positive bacteria predominate among sepsis case with *Staphylococcus aureus* [5] and *Streptococcus pneumoniae* being the most frequently bacteria isolated, closely followed by group B *Streptococcus* (GBS) [6].

Vertical transmission from the mother plays an important role in early neonatal sepsis. Newborns may be infected during labour as they pass through the birth canal, or during the first hours/days of life through close physical contact with the mother, if, as is commonly the case in SSA, the mother carries pathogenic bacteria in the nasopharyngeal tract or breast milk [5, 8–10].

Azithromycin (AZI) is a macrolide with a wide antimicrobial spectrum; it is effective against organisms such as macrolide-susceptible *Staphylococcus* species and *Streptococcus* species [11]. AZI is currently licensed for use in infants aged >6 months and is used to treat a wide range of infections, including those occurring during pregnancy [12, 13]. Data on the effect of treating pregnant women with up to 2 g of AZI for prevention of malaria and preterm birth are available [12–16]. Although scarce information exists, AZI appears to be excreted in human milk at a very low concentration [17]. According to a case report, an infant who is exclusively breastfed would receive approximately 1/10 or 1/20 of the dose recommended in infants ≥6 months old [18]. Thus far, there are limited safety data on AZI in neonates. Two trials among hospitalized neonates (one including 111 low birth weight neonates admitted to hospital and the other involved 2400 infants including neonates) have not shown severe adverse reactions [19, 20]. In another published study, AZI was given to 58 newborns as prophylaxis for pertussis [17] and only mild gastrointestinal adverse reactions were observed [16]. No additional adverse events were observed in the same study among newborns from AZI treated mothers [17].

Orally administered AZI is widely distributed throughout the body. In pregnant women, after one oral dose, peak plasma concentrations are attained within 6 h; myometrium concentrations reach high levels within this time [21]. The elimination half-life from plasma and tissues is 2–4 days. Some published literature show adverse reactions (i.e. gastrointestinal, headache) or allergic reactions (i.e. rash or swelling).

As part of the WHO-recommended trachoma control strategy, mass AZI treatment campaigns have been carried out in several trachoma endemic countries [22, 23], including The Gambia [24]. The additional benefits of these campaigns include decreased nasopharyngeal bacterial carriage [25], and childhood morbidity and mortality [26].

In The Gambia, the prevalence of AZI resistance after these mass AZI treatment campaigns was very low. For example, among *S.pneumoniae* isolates collected 6 to 30 months after treatment, resistance ranged between 0.3 and 0.9 % [25]. The relevance of acquired macrolide resistance for bacterial infections is uncertain but likely to be minimal in settings like the Gambia (and other African countries) where macrolides are very rarely used as empirical antibiotic therapy. In addition, there is evidence elsewhere from patients with cystic fibrosis receiving long term AZI, that although macrolide resistance develops in *S. aureus* among treated patients, these resistant organisms are not transmitted to their close family contacts [27].

New interventions to decrease neonatal mortality are urgently needed. If the mother is an important source of bacterial transmission to the newborn, then an intervention that reduces bacterial infections in the mother may prevent bacterial transmission and neonatal sepsis. In this study, we evaluate the impact of one oral dose of AZI given during labour on the asymptomatic bacterial carriage in the newborn baby as well as the mother. If successful, this simple intervention could be easily implemented at even the most remote health facilities. It has the potential to achieve wide coverage in SSA where low-cost interventions to reduce neonatal mortality are urgently needed.

Methods/design

Study objectives

The primary objective of the trial is to determine the impact of one oral dose of 2 g of AZI, given during labour, on the newborn's nasopharyngeal bacterial carriage at the age of 6 days for at least one of three pathogens: *S.aureus*, GBS and *S. pneumoniae*.

Secondary objectives are to determine the impact of the intervention on bacterial carriage in the nasopharyx of the baby, and in the vaginal tract and breast milk of the mother at different time points within the first 4 weeks after delivery. The impact of AZI on purulent conjunctivitis and ocular *C. trachomatis* infection will also be assessed.

To evaluate the safety of the intervention on mothers and newborns we monitored and assessed adverse events (AE). Special attention was paid to hypertrophic pyloric stenosis (HPS) in the newborn which is usually reported as projectile vomiting.

Study endpoints

The primary endpoint is the prevalence of nasopharyngeal carriage of the newborn at the age of 6 days for any of the following bacteria: *S.aureus*, GBS and *S.pneumoniae*.

Secondary endpoints include:

- Prevalence of nasopharyngeal bacterial (*S.aureus*, GBS and *S.pneumoniae*) carriage of the newborn at different time-points during the first months of life.
- Prevalence of nasopharyngeal, vaginal and breast milk bacterial carriage (*S.aureus*, GBS and *S.pneumoniae*) of the mother at different time-points during the first month after delivery.
- Prevalence of non-susceptibility to macrolides among bacteria (*S.aureus*, GBS and *S. pneumoniae*) isolated from the clinical samples collected from the newborns and their mothers during the first month after delivery.
- Proportion of newborns with at least one episode of purulent conjunctivitis and ocular *C. trachomatis* infection within the first week of life, the first 4 weeks of life and during the 8 weeks of the follow-up period.

We also measured AZI concentrations in the breast milk of the first 40 women recruited into the trial at two time points during the first week of life . Other endpoints not initially included in the protocol are weight gain during the first 8 days of life and the time from BCG vaccination to the appearance of a scar.

As safety endpoints, we included the number of solicited and unsolicited AEs in mothers and newborns observed during the first 6 days after treatment; and number of AEs in mothers and newborns reported during the first 8 weeks after treatment.

Trial design

This is a Phase III, double -blind, placebo controlled randomized clinical trial in which pregnant women in labour attending study health facilities centre were randomized to either a single dose of 2 g of oral AZI or placebo (ratio 1:1).

Blinding and Un-blinding

The packaging and labelling of the interventional medical product (IMP) was conducted by IDIFARMA. AZI and placebo were provided as tablets packed in blisters. The randomisation list was created by an independent statistician/data manager and IDIFARMA numbered the blisters according to the list. One blister pack of IMP contained four tablets of 0.5 g of AZI or placebo (2 g in total). The active drug and the placebo looked identical and were not identifiable.

Sealed envelopes containing the individual treatment allocation were kept at the study site for emergency cases. The code could be broken for individual participants in cases of AE for which the investigational product needed to be known. The code was kept for the entire study period by the independent statistician of the Data Safety Monitoring Board (DSMB) and will be broken only after the database lock.

Study setting

The study was conducted in a peri-urban area situated on the western margin of The Gambia, approximately 20 km from the capital Banjul. The population includes the main ethnic groups in The Gambia, engaged in a wide range of occupations, including working for government services, liberal professions and trading. The climate of the area is typical of the sub-Sahel region, with a long dry season from November to May and a short rainy season between June and October. Much of the population is illiterate [2].

The study was carried out in the Jammeh Foundation for Peace Hospital (JFPH), a government-run health centre located approximately 8 km from Fajara where the main MRC laboratories are based. This health centre serves a local community of more than 50,000 inhabitants and a large catchment area with several neighbourhoods; and manages approximately 5,000 deliveries per year (Fig. 1).

Ethical considerations

All study mothers had signed consent during their prenatal visits before being enrolled into the trial. A local safety monitor (LSM) and a Data Safety Monitor Board (DSMB) reviewed all the SAE during the course of the trial, and the trial was monitored by an independent clinical trials monitor. The study was approved by the joint Medical Research Council (MRC)/Gambia Government Ethics Committee.

Sensitization and consenting

Community sensitization

Initial sensitization of community leaders was done during an Open Day at JFPH. During the process of community sensitization, the study field team asked the community leaders to identify literate six people from the community who would be willing to act as impartial witnesses.

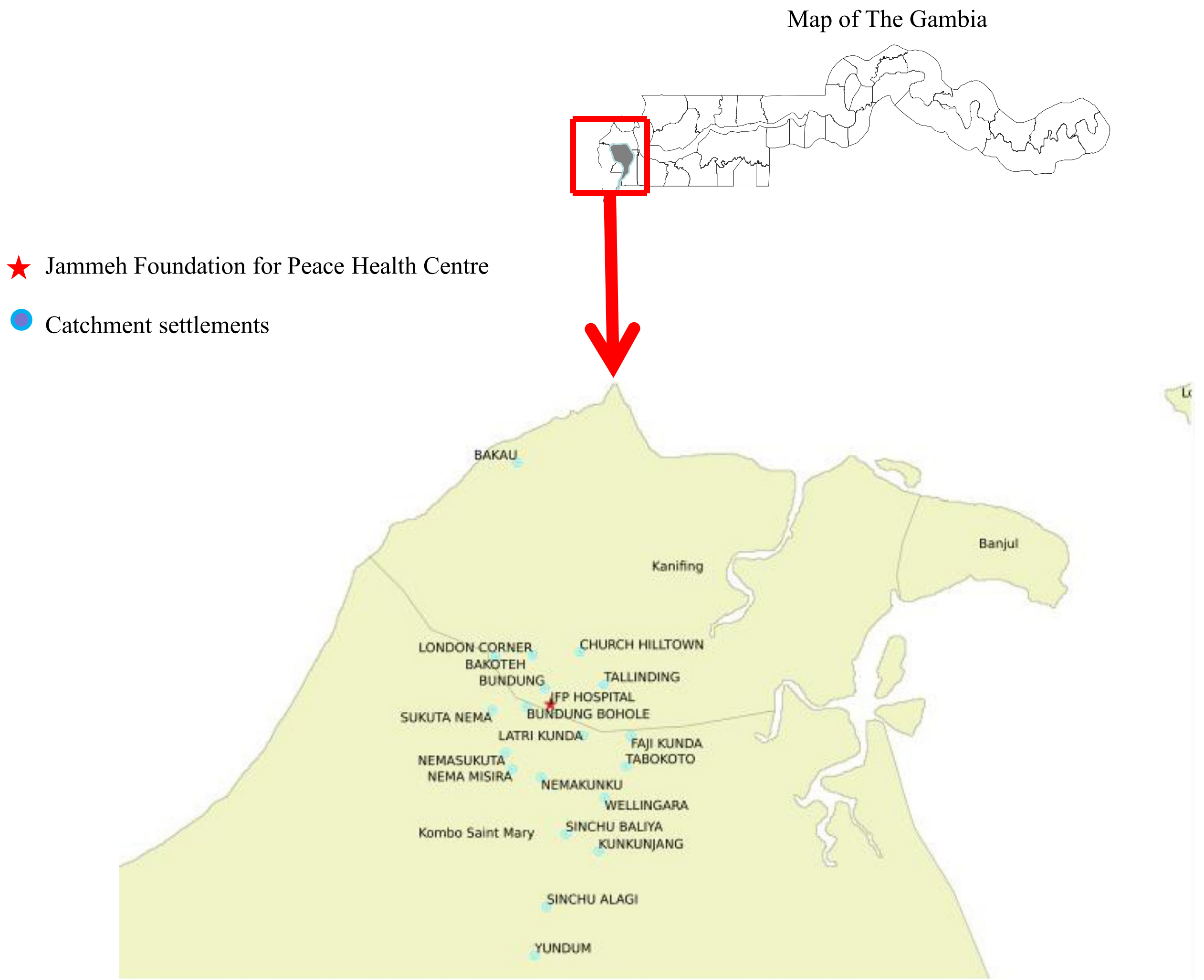

Fig. 1 Study site

Identification of pregnant women

A field worker explained the study to pregnant women attending the JFPH for antenatal care and planning to deliver their babies there. The explanation occurred soon after the women had received the standard health education talk from the nursing staff in charge of the antenatal clinic.

Women sensitization

Interested women were invited to the field team office where the study explained in their preferred language. After obtaining the woman's agreement, a unique sensitization sticker was placed on her antenatal card. During the course of the study, awareness of the nature of the study, increased steadily as the message had spread within the community.

Consenting

After sensitization, an individual consenting process was started. The informed consent form (ICF) could only be signed at JFPH. Mothers either signed the ICF or, if they were illiterate, thumb printed it. An impartial witness was present during the consenting process for illiterate women.

Women were encouraged to ask questions during consenting. If the field worker could not answer questions raised by the women, either the nurse coordinator or a study clinician was then involved in the process. The woman's understanding of the information was tested using an informed consent understanding tool questionnaire with 13 questions. A maximum of two attempts were allowed; the consenting process was stopped if after a second attempt at least one of the questions was not correctly answered (Fig. 2). The completed ICF was

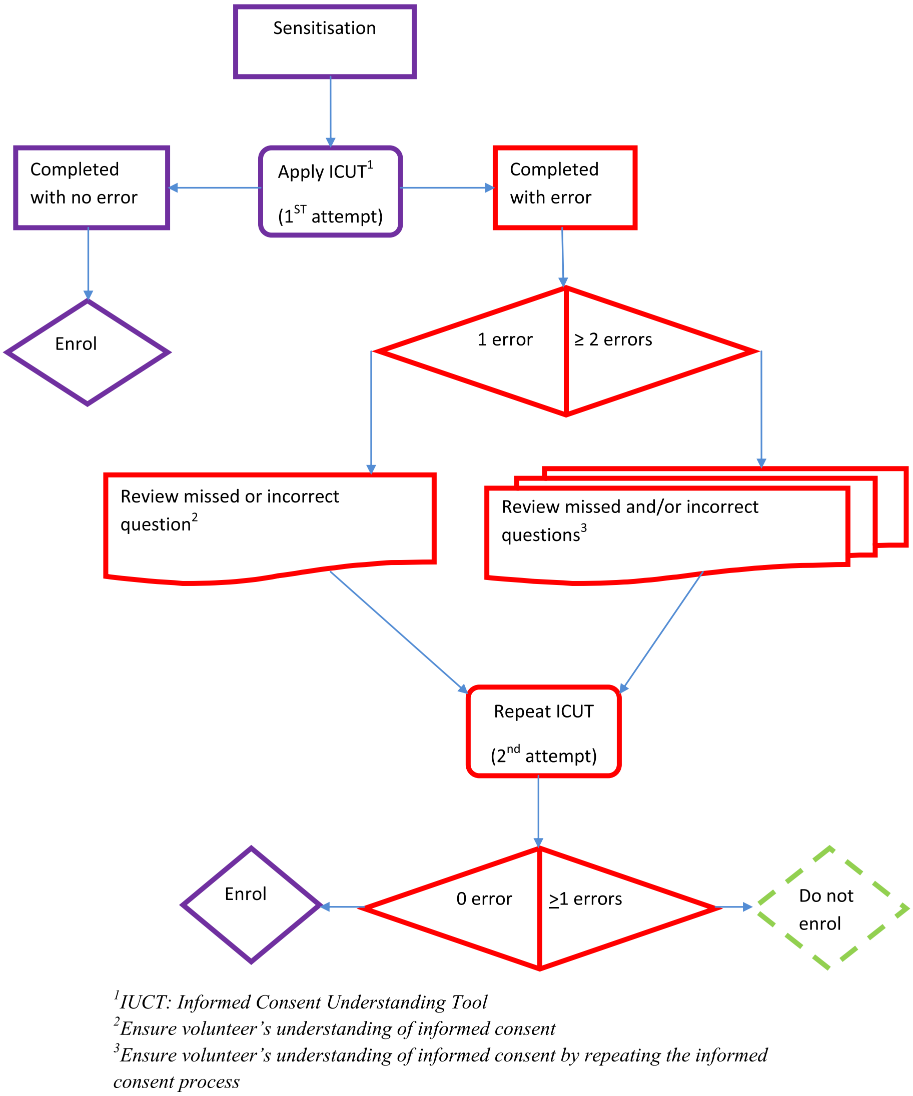

Fig. 2 Sensitisation and Informed Consent Understanding Tool Process

attached to the woman's antenatal card and a copy was kept securely in the study office.

Pregnant women were informed that signing/thumb printing the ICF did not automatically result in being enrolled in the study; this occurred only if the woman delivered at the study site at the time the study nurses were available to screen, randomize and treat them.

Screening, recruitment and randomization

Screening

When women presented to the labour ward in labour, a study nurse confirmed she had signed consent and assessed the continued willingness of the women to participate in the study. Subsequently, eligibility was determined using the criteria detailed below.

- Inclusion criteria: Pregnant women in labour aged 18 to 45 years presenting to the JFPH for delivery
- Exclusion criteria
  - Known HIV infection
  - Any chronic or acute condition that might interfere with the study as judged by the research clinician
  - Planned travel out of the catchment area during the 2 months after delivery (follow-up period)
  - Planned caesarean section
  - Known required referral
  - Known multiple pregnancy
  - Known severe congenital malformation of the baby
  - Intrauterine death confirmed before randomization

– Known allergy to macrolides
– Intake of antibiotics in the week before randomization

Recruitment

Once eligibility was determined, baseline or pre-intervention samples were collected from the mother [nasopharyngeal swab (NPS) and vaginal swab].

Randomization

IMP were labelled from 001 to 830. A random control digit was added to the identification number after a backslash – e.g. 001/1, to help detect transcription errors. The IMPs were assigned consecutively and in ascending order. The time when the drug was taken was recorded. The randomization number on the specific blister was written in the spaces (– – – / –) provided on the particular page of the case report form (CRF). Any vomiting (if it occurred), was documented including the timing and whether an IMP tablet was seen in the vomitus.

Health centre discharge, follow up and sampling (Table 1 and Fig. 3)

Women were kept under close observation by study nurses. The JFPH nurses conducted the delivery. When the baby was born, the study nurse on duty collected the NPS within 6 h of birth. A clinical examination of both mother and baby were carried out by one of the study clinicians before discharge (Table 1), which occurred 6 to 24 h (mostly within 6–10 h) after delivery if both were clinically stable and there were no serious adverse events (SAE). Then, participants were given a Participant Identification (ID) card for further identification.

Home visits were conducted for each mother/baby pair for 8 weeks. In the first week, a nurse followed-up both mother and baby with daily recording of information on the health status of the pair and any adverse events (AE). During this week, samples were collected on days 3 and 6, including NPS from mother and baby and breast milk from the mother (Table 1).

Between the 8 and 10 days after the delivery, both mother and baby presented at the study site where the research clinician evaluated them and a vaginal swab was collected from the mother (Table 1). At that point, the babies presented at the infant welfare clinic to receive their BCG, OPVo, and Hepatitis B vaccines.

During the next 7 weeks, a field worker followed up the mother/baby pair. At weeks 2 and 4, breast milk samples were collected from the mothers and NPS from both mothers and babies (Table 1). If the baby was noted to have eye discharge during any of the visits, an eye swab was collected and clinical information recorded. Treatment was given appropriately.

In the advent of any symptoms or signs of illness during home visits, the field worker called the nurse coordinator who, if necessary, asked the mother and baby to go to the study site to be assessed by the nurse coordinator or his assistant or when indicated, by the study clinicians.

If during the follow-up period, either mother or baby needed antibiotics, samples were collected in lieu of the next scheduled sample and participants were started on antibiotics. If a mother was on antibiotics, no more samples were collected from her or the baby. If the baby was on antibiotics, subsequent samples were still collected from the mother.

Table 1 Summary of the study activities including follow up visits and sampling

Visit number	Pre-intervention	Post-intervention						
	Day 0 (delivery)	Day 0 (delivery)	Day 1 to 6	Week 1 (days 8–10)	Week 2	Week 3	Week 4	Week 5 to 8
Window period	—	——	1 day	Day 6 to 13	−2/+4 days	−3/+3 days	−3/+3 days	−3/+3 days
Health Centre visit	X	X		X				
Home follow-up visits			X		X	X	X	X
Informed consent	X							
Review inclusion/exclusion criteria	X							
Adverse events	X	X	X	X	X	X	X	X
NPS mother	X		X[a]		X		X	
NPS newborn		X	X[a]		X		X	
Breast milk sample			X[a]		X		X	
Vaginal swab	X			X				

[a]Only days 3 and 6

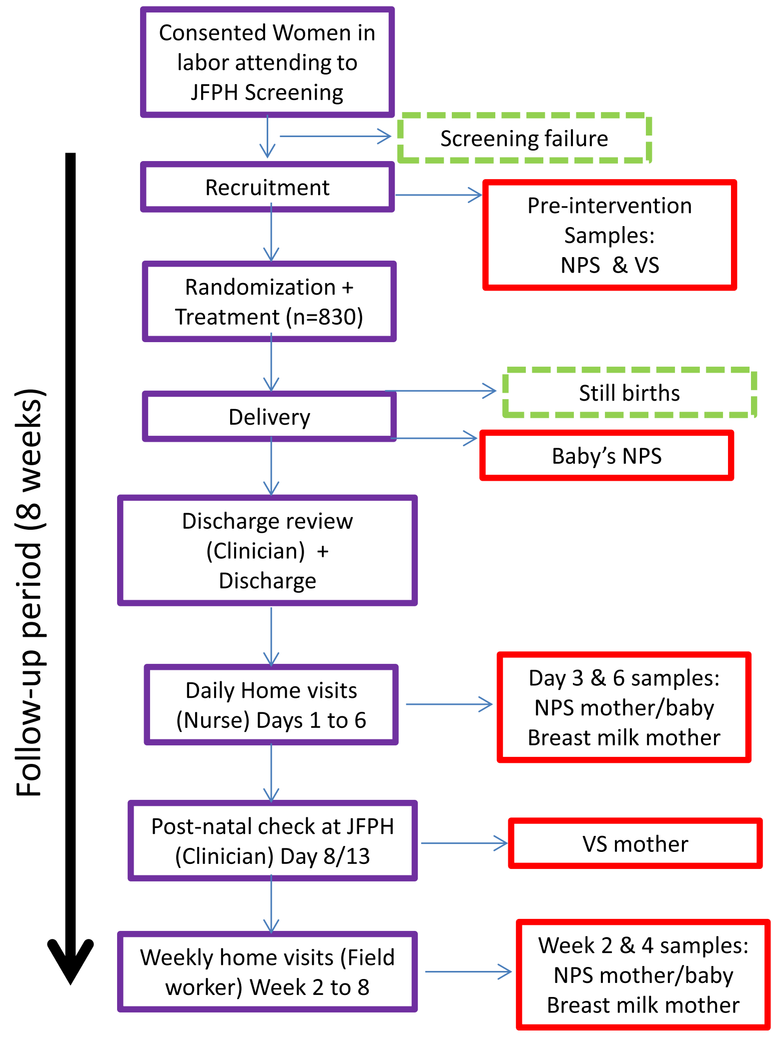

Fig. 3 Overall study approach

The last field worker visit occurred 8 weeks after birth. This marked the study termination date for the mother/baby pair.

Safety considerations and serious adverse events (SAEs)

Safety of the trial

A local safety monitor (LSM) and a Data Safety Monitor Board (DSMB) who reviewed all the SAE during the course of the trial was established.

Referral procedures for SAE

Any AE for either a mother or a baby, was captured in the CRF. When a study baby visited JFPH with any illness or called the nursing team, s/he was initially seen by the nurse on duty who determined the need for the baby to be reviewed by the study physician at the health centre. If referral was needed, the clinician recorded and instituted initial management of the child's condition before subsequently referring him/her to the MRC ward in Fajara or the Edward Francis Small Teaching Hospital (EFSTH) in Banjul. Study related procedures (such as daily/weekly visits) continued as per protocol.

Hypertrophic pyloric stenosis (HPS)

Because of the lack of information on breast milk trespass of AZI and effects of AZI in the newborn, there was a risk of HPS. Field workers and nurses were trained to recognise HPS through abdominal palpation and auscultation. In the event of a suspected HPS case or severe vomiting reported by the mother, study staff were advised to immediately inform the study clinicians and PI. Subsequently, the child was to be transferred to the MRC Ward in Fajara for a thorough clinical assessment. The following steps were put in place:

– Diagnosis by the research clinician using clinical judgement and conducting laboratory (electrolytes) and imaging studies such as abdominal x-ray to eliminate other potential diagnoses.
– If necessary, transfer to the EFSTH, in Banjul, where surgical review and additional assessment could be performed, including ultrasound if deemed necessary to confirm HPS diagnosis.

If the diagnosis of HPS is confirmed, the definitive treatment is surgical.

Samples and laboratory methods

Sample collection

Clinical or field staff who received training on sample collection as per study protocol were involved in the collection of the following samples:

Nasopharyngeal Swabs (NPS) were collected by inserting a calcium alginate (Expotech USA Inc) swab into the posterior wall of the nasopharynx. The swab was rotated and left in the nasopharynx for approximately 5 s. The inoculated swab was placed immediately into a vial containing skim milk-tryptone-glucose-glycerol (STGG) transport medium and then into a cold box before transporting to the MRC Laboratories within 8 h, in accordance with the WHO protocol for evaluation of bacterial carriage [28].

Low Vaginal swabs (VS) were collected by inserting a sterile cotton swab (Sterilin Ltd, UK) 2–3 cm into the vagina and rotating the swab with a circular motion, leaving it in the vagina for approximately 5 s. The inoculated swabs were then placed immediately into the vials containing STGG and put in a cold box before being transferred to the MRC Laboratories within 8 h.

Breast milk samples

The nipple and areola of the breast from which milk was taken was disinfected using sterile cotton soaked with 0.02 % chlorhexidine. Mothers were then asked to manually express their milk. The first 0.5 mL were discarded. The following 1-2 mL was collected in another sterile plastic bijoux bottle put in a cold box and transferred to the MRC Laboratories within 8 h.

Eye swabs were collected using a sterile cotton swab (Sterilin Ltd, UK). The lower eyelid was pulled down to expose the conjuctival sac inside the lower eyelid. The sterile cotton swab was rolled on the conjuctival sac, parallel to the cornea to avoid injury. The inoculated cotton swabs were immediately returned to its container, put in a cold box and transported to the MRC Laboratories within 8 h.

Bacteriological analysis for NPS, VS and BM samples

Samples received in the lab were vortexed for 20 s before being stored at −70 °C for subsequent processing in batches. During processing, samples were allowed to thaw on ice. Each vial was then vortexed briefly in order to homogenise the medium and 50 μl was dispensed onto gentamicin blood agar (GBA) (CM0331 Oxoid, UK +5 % sheep blood), mannitol salt agar (MSA)(CM0085 Oxoid, UK) and crystal violet blood agar (CVBA)(CM0085 Oxoid, UK +0.02 % crystal violet) for selective isolation of *S. pneumoniae*, *S. aureus* and GBS respectively. Each inoculum was streaked onto four quadrants in order to semi-quantitatively determine the bacterial load [29]. Colony density was determined by recording growth of alpha-haemolytic colonies (GBA plates), yellowish dome shaped colonies (MSA plates) and beta haemolytic colonies (CVBA plates) as 4+ if >10 colonies are present in quadrant 4, 3+ if there are <10 colonies in quadrant 4 and > than 10 colonies in quadrant 3, 2+ if there are <10 colonies in quadrant 3 and > than 10 colonies in quadrant 2, and 1+ if there are less than < 10 colonies in quadrant 1.

Streptococcus pneumoniae

After 20–24 h incubation at 37 °C with 5 % CO_2, GBA plates were examined for typical alpha haemolytic colonies. A single colony was picked and sub-cultured onto another Blood agar (BA) to obtain pure growth and screened for optochin susceptibility [28]. Zones of inhibition ≥14 mm indicate susceptibility, 7–13 mm were intermediate and <7 mm indicate resistance to optochin (WHO protocol). Optochin susceptible isolates were confirmed pneumococci; those of intermediate susceptibility were confirmed by the bile solubility test. Those that are optochin resistant were considered to be species other than pneumococcus.

Pneumococcal latex serotyping

Pneumococcal serotyping was performed as described previously [30]. In brief, pneumococcal cell suspension was made in 2 ml normal saline (1.0 McFarland) from an overnight culture. 50 μl of the cell suspension was dispensed into ten wells of the serotyping tray. An equivalent amount of each of the main group latex antisera (A, B, C, D, E, F, G, H, I and Omni) was added to the wells. The mixture was rugged for a maximum of 2 min and agglutination(s) observed in the wells. The procedure was repeated with the sub-groups of the main groups in which agglutination was observed. The subgroups that reacted were further typed with the corresponding subtypes and the agglutinations noted. The resulting serotype was determined with the aid of a chart (Statens Serum Institute, Denmark).

Staphylococcus aureus

After forty-eight hour incubation at 37 °C, MSA plates were examined for typical staphylococci colonies. Pale to

golden yellow domed shaped colonies 1–2 mm in diameter were cultured onto Blood agar to obtain pure growth. Catalase test was performed on all suspected colonies and catalase positive colonies were further tested using the coagulase test. Isolates positive for coagulase test were confirmed to be *S. aureus.*

Group B streptococci

After 20–24 h incubation, presumptive beta-haemolytic colonies were streaked onto BA agar to obtain pure growth. Catalase test was performed using hydrogen peroxide to quickly differentiate the presumptive *Streptococci* from *Staphylococcus species.* Beta-haemolytic and catalase negative isolates confirmed to be streptococci were grouped using the Streptex grouping kit (Remel R30950501) and ultimately reported as A, B, C, D, F or G.

Antibiotic susceptibility testing

Between 3 and 5 well-isolated colonies of similar morphological appearance were transfered into 2.5 ml physiological saline until visible turbidity equal to 0.5 % McFarland's Standard was obtained. A sterile swab was then immersed into the suspension and then streaked evenly over the surface of the Mueller Hinton agar (MHA) with 5 % sheep blood (*S. pneumoniae* and GBS) and MHA without blood (*S. aureus*) in three directions, rotating the plate 90 °C to ensure even distribution.

Azithromycin resistance was screened by disk diffusion (15 μg azithromycin disc) and those intermediated or resistant were determined by E-test following the clinical and laboratory standard institute (CLSI) 2014 guidelines for the performance of test and interpretation of results: (i) *S.aureus* isolates with E-test values ≥8 μg/ml were reported as resistant whilst values <8 μg/ml ≥ 4 μg/ml as intermediate resistant; (ii) *S. pneumoniae*/GBS isolates with E-test values ≥2 μg/ml were reported as resistant whilst values <2 μg/ml ≥ 1 μg/ml as intermediate resistant.

Molecular analysis for ocular swabs

DNA was extracted from all ocular swabs using the QIAamp DNA Mini Kit (QIAGEN, Hilden, Germany) according to the manufacturer's instructions. The presence of *C. trachomatis* DNA was assayed for using the FTD Vaginal Swab kit (Fast-track Diagnostics, Junglinster, Luxembourg) on a RotorGene 6000 real-time PCR cycler (QIAGEN, Hilden, Germany). Samples were further tested for the presence of *C. trachomatis* DNA using the droplet-digital PCR method as described by Roberts et al. [31].

Azithromycin measurement

AZI levels were measured using a validated liquid chromatography-mass spectrometry (LC-MS) method using a triple quadrupole MS (8030 plus, Shimadzu, Kyoto, Japan) and a deuterated internal standard. In brief, 20 μL samples of milk were spiked with internal standard and extracted based on methodology developed for plasma samples (Salman et al. Antimicrob Agents Chemother 2010;54: 360–366). After addition of ammonium hydroxide and methanol, vortex mixing and freezing, the thawed sample was centrifuged and the organic supernatant mixed with water and an aliquot injected onto an Agilent Eclipse plus C18 column (Agilent Technologies, Santa Clara, CA). MS quantitation was performed in multiple reaction monitoring mode using an ESI+ ion source. Matrix effect, process efficiency and absolute recovery for AZI and AZI-d_3 were within acceptable ranges (means 92.5 to 107.9 %). Intra- and inter-day relative standard deviations were 3.1–5.2 and 6.6–9.7 %, respectively, at AZI concentrations between 100 and 5000 μg/L. Mean accuracy was between 94.5 and 103.8 % over the same concentration range. The limits of quantitation and detection were 5 μg/L and 2.5 μg/L, respectively.

Data considerations

Data handling and record keeping

Each participant pair had a unique identification (ID) number. All data recorded on individuals used the anonymous ID. All forms with subject names is kept in a locked cabinet, when not in use. Clinical data is stored separately from that containing personal information. Data will be stored for at least 10 years. The informed consent forms and source documents will be stored, after study completion, in a confidential archive; they will be stored separately from the study documents in the access-restricted archive at the MRC Unit.

Some sections of the CRFs act as source data for the trial and are completed directly by the relevant research personnel. A few sections, such as vaccination, are transcribed from the Infant Welfare Card for the newborn. Other information from the mother and the baby (such as age of the mother or birth weight of the baby) was transcribed from the antenatal card. Other source data include: the structured clinical notes at enrollment, discharge and post-natal visit, and the structured information collected from field workers/nurses during the weekly home visits (week 2 to week 8). At each visit, a CRF was completed by the relevant study team member. In addition, laboratory forms were also completed for any sample collected as part of the study from either the mother or the baby.

CRF data is entered into an *OpenClinica* (www.openclinica.com) database using double data entry and verification. Laboratory data have been recorded on lab forms designed specifically for the study and entered into a separate site within the *OpenClinica* study database. Screening information was entered in another sub-

database within the main *OpenClinica* database. Screening records are uniquely identified by the sensitization number, which is issued at the time of sensitization. Consistency checks have been performed during data entry and outliers and missing data checked against the original forms then subsequently amended in the database.

The PI and sponsor maintain appropriate medical and research records for this study in compliance with applicable Good Clinical Practice (GCP), regulatory and institutional requirements. Authorised representatives of the sponsor, the ethics committee or regulatory bodies may inspect all documents and records maintained by the investigator. The PI or designee ensures access to facilities and the records.

The PI or designee documented and explained any deviation from the approved protocol and reported them to the sponsor and ethics committee in accordance with the MRC Unit's standard procedures.

At study closure, all items on the following checklist will be checked prior to locking the database:

- All expected study subjects have been entered
- Checks for any duplicate subjects is negative
- SAE reconciliation has been completed
- Protocol Deviation reconciliation has been completed.
- All final cleaning checks have been performed and all outstanding issues resolved
- Approval for database lock has been obtained from the Head of Data Management (HoDM), the statistician, and the PI.
- Database access rights have been removed.

The randomisation list will be kept by the statistician of the DSMB until the blinded results of the statistical analysis are available. The study site will retain a set of envelopes containing the allocation of each subject so that individuals can be unblinded for safety reasons without accessing the randomization list.

Trial monitoring

A clinical trials monitor independent from the investigator team monitored the trial. The monitor conducted an initiation visit to the site before the trial started to verify and document that all study staff were adequately informed about the study and that the IMP to be used had been received and stored accordingly on site.

During the course of the trial, interim monitoring visits took place at the health centre, the IMP storage facility, the site office and the laboratories.

During the visits, the monitor carried out a quality control of trial progress in respect to protocol and operating guidelines, data collection, completion of consent forms, completion of study documents, SAE reporting, and samples and IMP management. Source data verification was conducted for all ICFs, eligibility criteria and SAEs.

The monitor inspected up to 10 % of participants CRFs for accuracy, completeness and consistency with source documents before data entry and reviewed the investigator's file.

At the end of each monitoring visit, the monitor discussed the findings with the study team and outlined what further actions should be taken.

The Monitor will conduct a close-out visit after all data queries have been resolved and the data base has been locked to ensure that all essential documents are completed and filed accordingly and are ready for archiving, that all IMPs have been accounted for and are returned to supplier or destroyed accordingly, and that all unused study materials are returned to suppliers or destroyed, as applicable.

The study team established, in addition, internal monitoring procedures to ensure review of all CRFs by study team members before any data was entered into the database.

Sample size rationale

The original sample size (415 mother/baby pairs per arm) assumed a 15 % loss to follow-up (i.e. 353 participants in each arm for whom the primary endpoint is recorded). Based on data from a previous study [32], bacterial carriage (*S.aureus*, GBS or *S.pneumoniae*) on day 6 was assumed to be 60 % in the control arm. According to these assumptions, the study has 98 % power to detect a 25 % reduction in bacterial carriage (GBS, *S.pneumoniae* or *S.aureus*) and 88 % power to detect a 20 % reduction.

Statistical analysis

A flow chart will be used to describe the number of mothers who were eligible, randomised and treated, and the number of samples obtained from mothers and children at each time point.

The adequacy of the randomisation procedure will be checked by comparing baseline characteristics of mothers (ethnicity, age, season enrolled, hours from treatment to delivery, mode of delivery, time to rupture of membrane, apgar score, multiple pregnancy) and newborns (sex, birth weight, gestational age) between arms. Some of these variables are measured post intervention, but are unlikely to be influenced by the intervention (time from treatment to delivery, mode of delivery, time to rupture of membrane, Apgar score). Prevalence of bacterial carriage in the mother pre-intervention will also be used to check comparability of baseline characteristics (NPS and vaginal swab).

The primary analysis will be based on intention to treat (ITT). We will include data on twins but will not adjust for the effect of clustering (the average cluster size will be close to 1 so the design effect will be negligible). A per protocol analysis will be conducted excluding samples from mothers who vomited less than 15 min after medication was taken, samples collected after the use of antibiotics, and samples arriving at the lab more than 8 h after collection.

The prevalence of bacterial carriage will be compared between arms at each time point (in NPS, vaginal swabs and breast milk). The prevalence of carriage will be presented separately for each species and combined (i.e., carriage of *S.aureus*, GBS or *S.pneumoniae*). In addition, carriage acquisition rates will be compared between arms for the periods 0–6 days and 7–28 days (acquisition will happen when a non-carrier will become carrier in the subseuqent sample). Ratios of prevalence and 95 % confidence intervals will be calculated for each of these comparisons, and Fisher's exact test will be used to compute p-values for the comparisons of prevalence.

A Poisson regression model will be used to assess the effect of the intervention on prevalence of carriage across all time points. The model will include time, baseline carriage status of the mother, mother's age, ethnicity, season, sex of the newborn, and gestational age. Robust standard errors will be used to account for the dependence between observations from the same individual as well as the model misspecification (carriage status is binary and therefore does not follow a Poisson distribution).

To calculate prevalence of carriage at day 3 and day 6 we will use data from samples (NPS newborns and mothers, breast milk) collected within 1 day of the scheduled visit. At 14 days we will use samples collected between 12 and 18 days, and at 28 days we will use samples collected between 25 and 31 days. For vaginal bacterial carriage at day 8–10 we will use samples collected between 7 and 13 days. Samples were not collected after children received antibiotics. Where a sample was collected in lieu of the missing sample (i.e., before antibiotics were given) this will be used in place of the missing sample, even if it was collected outside the appropriate window period.

We will present analyses based on the available data. For the primary outcome we will conduct two additional analyses using the ITT cohort. First, a subgroup analysis in women who gave birth 2 h or more after taking the drug. And second, a sensitivity analysis using multiple imputation of missing data. In the imputation model, bacterial carriage in missing samples will be imputed from data on bacterial carriage at other time points and baseline demographic data (age and ethnicity for mothers, and sex and birth weight for newborns).

Changes in prevalence of bacterial carriage over time will be plotted for NPS in mothers and newborns, and breast milk. The plots will include point-wise 95 % confidence intervals for each prevalence estimate.

The numbers and rates of adverse events, serious adverse events (overall and specifically with a diagnosis of neonatal sepsis), purulent conjunctivitis, Chlamydial conjunctivitis, and deaths in newborns will be reported by trial arm. Rates of adverse events and serious adverse events will be reported in mothers. Rates will be compared between trial arms using Poisson regression, and robust standard errors will be used to allow for heterogeneity in the rates between individuals.

Discussion

Neonatal deaths, estimated at approximately 4 million annually, represent up to 40 % of all child fatalities with 3 out of 4 of these neonatal deaths occurring during the first week of life with maximum risk during the first 3–4 days. Interventions designed to impact on child mortality should target this vulnerable age group.

Sepsis causes up to 1 out of 3 neonatal deaths. Many neonatal casualties associated with sepsis are preventable when appropriate interventions are put into place. In resource-rich countries, GBS infections were substantially reduced with antibiotic-based prevention strategies. For example, chemoprophylaxis with ampicillin or penicillin in the USA reduced the incidence of early onset neonatal sepsis caused by GBS from 1.8 per 1,000 live births in the early 90's to 0.28 per 1,000 in 2010 [33]. In these countries, when a screened woman is identified as a GBS vaginal carrier, she is treated during labour intravenously with antibiotics [33, 34]. To reach maximum effectiveness, treatment should last at least 4 h before delivery [33, 34]. Maternal antibiotic treatment during labour blocks bacterial transmission to the newborn at birth. Because the newborn does not become infected, s/he is not at risk of developing early neonatal severe disease. Before GBS screening was introduced in USA and other developed countries, the great majority of neonatal sepsis, especially those occurring during the early neonatal period, were caused by this bacterium.

To roll-out a potentially successful intervention to developing countries within Africa and elsewhere, main epidemiological and contextual differences between the regions need to be considered. Unlike developed countries, several bacteria are associated with early and late neonatal sepsis in the African continent and intravenous antibiotics are generally unavailable. Approximately two thirds of neonatal sepsis cases in Africa are caused by gram positive bacteria [9] and in some countries gram positive bacteria represent up to 80 % of all sepsis during the first month as well as the first week of life. Due to costs, limited infrastructure and diagnostic facilities,

screening programmes are difficult to implement in SSA and other developing regions. Still, screening may not be needed where at least half of pregnant women are vaginal carriers of one of the most common bacteria associated with neonatal sepsis [35]. In Africa, other known risk factors for early neonatal sepsis, such as preterm or low birth weight [7] and premature rupture of membranes or prolonged labour [36], are also common.

The relevance of acquired macrolide resistance for outcome of episodes of clinical infection is uncertain, but is likely to be minimal in a setting like the Gambia and other African countries where macrolides are very rarely used as empirical antibiotic therapy. Also, previous studies in The Gambia show that after mass AZI campaigns for trachoma control, prevalence of AZI resistance among *S.pneumoniae* isolates was re-establisehd to baseline levels (<1 %) [25]. In addition, there is evidence elsehwere from patients with cystic fibrosis receiving long term AZI, that although macrolide resistance develops in *S. aureus* among treated patients, these resistant organisms are not transmitted to their close family contacts [27].

In this proof-of-concept study, we propose an intervention that would be easily implementable in developing countries. We treat women in labour attending a health facility with one dose of AZI, an oral antibiotic that is cheap and does not need specific storage conditions (can be kept at room temperature), and we will assess whether this antibiotic decreases the risk of bacterial colonization in the baby during the neonatal period (primary endpoint during the first week of life or early neonatal period) and the mother during the same period. The hypothesis that motivates our intervention is that the mother is the main source of bacterial transmission to the baby. If the intervention is successful in proving our hypothesis, then we will design several additional studies. First, we will assess the impact of the intervention on severe clinical endpoints such as neonatal sepsis and mortality in the baby and puerperal sepsis in the mother. If this is shown to be of benefit, we will then test the intervention in the context of home delivery, as in Africa, many women deliver at home attended by traditional birth attendants.

Trial status

At the time of submission of this manuscript, ethical approval had been obtained and recruitment had finished. Laboratory work and data cleaning was ongoing.

Competing interests

All authors declare no competing interest.

Author's contributions

AR and UDA conceived and designed the study. AR drafted the protocol and wrote the manuscript and UDA contributed significantly in the final version of both. CO, BC, AB developed and adapted the field and laboratory work and made contributions to the development of the manuscript. SB conducted the eye swabs analysis. CB provided statistical input to the study. TD conducted the azithromycin measurement. JM, RB and BK contributed to protocol and the manuscript. All authors read and approved the final manuscript.

Acknowledgements

We thank the mothers and their newbors who participated in our study. We are especially grateful to the study field team (lead by Edrissa Sabally and Omar Jarra), the laboratory team (Isatou Cox and colleges) and the data team (Bai Lamin Dondeh, Kodou Lette and Maimuna Sowe and the data entry team). Our thanks also extend to the CEO of the Jammeh Foundation for Peace Hospital Team (Kebba Manneh) and his team. We are also thankful to our internal monitor (Vivat Thomas), the local safety monitor (Dr Aderonke Odutola) and the DSMB team chaired by Prof Daniel Chandramohan (with Prof Brian Greenwood and Drs Maria Quigley, Stephen Howie and Hannah Blencowe).

Financial disclosure

The MRC Unit in The Gambia receives core funding from the MRC UK. This trial is jointly funded by the UK MRC and the UK Department for International Development (DFID) under the MRC/DFID Concordat agreement (reference number MR/J010391/1).

Author details

[1] Medical Research Council Unit, Fajara, The Gambia. [2]London School of Hygiene and Tropical Medicine, London, United Kingdom. [3]School of Medicine and Pharmacology, University of Western Australia, Fremantle Hospital, Fremantle, Western Australia, Australia.

Received: 21 November 2014 Accepted: 10 November 2015
Published online: 19 November 2015

References

1. Hill K, You D, Inoue M, Oestergaard MZ. Child mortality estimation: accelerated progress in reducing global child mortality, 1990–2010. PLoS Med. 2012;9:e1001303.
2. Jasseh M, Webb EL, Jaffar S, Howie S, Townend J, Smith PG, et al. Reaching millennium development goal 4 - the Gambia. Trop Med Int Health. 2011; 16:1314–25.
3. Lawn JE, Cousens S, Zupan J. 4 million neonatal deaths: when? Where? Why? Lancet. 2005;365:891–900.
4. Liu L, Johnson HL, Cousens S, Perin J, Scott S, Lawn JE, et al. Global, regional, and national causes of child mortality: an updated systematic analysis for 2010 with time trends since 2000. Lancet. 2012;379:2151–61.
5. Seale AC, Mwaniki M, Newton CR, Berkley JA. Maternal and early onset neonatal bacterial sepsis: burden and strategies for prevention in sub-Saharan Africa. Lancet Infect Dis. 2009;9:428–38.
6. Waters D, Jawad I, Ahmad A, Luksic I, Nair H, Zgaga L, et al. Aetiology of community-acquired neonatal sepsis in low and middle income countries. J Glob Health. 2011;1:154–70.
7. Schrag SJ, Cutland CL, Zell ER, Kuwanda L, Buchmann EJ, Velaphi SC, et al. Risk factors for neonatal sepsis and perinatal death among infants enrolled in the prevention of perinatal sepsis trial, Soweto, South Africa. Pediatr Infect Dis J. 2012;31:821–6.
8. Chatzakis E, Scoulica E, Papageorgiou N, Maraki S, Samonis G, Galanakis E. Infant colonization by Staphylococcus aureus: role of maternal carriage. Eur J Clin Microbiol Infect Dis. 2011;30:1111–7.
9. Rudan I, Theodoratou E, Nair H, Marusic A, Campbell H. Reducing the burden of maternal and neonatal infections in low income settings. J Glob Health. 2011;1:106–9.
10. Suara RO, Adegbola RA, Baker CJ, Secka O, Mulholland EK, Greenwood BM. Carriage of group B Streptococci in pregnant Gambian mothers and their infants. J Infect Dis. 1994;170:1316–9.
11. Drew RH, Gallis HA. Azithromycin–spectrum of activity, pharmacokinetics, and clinical applications. Pharmacotherapy. 1992;12:161–73.
12. Nosten F, McGready R, D'Alessandro U, Bonell A, Verhoeff F, Menendez C, et al. Antimalarial drugs in pregnancy: a review. Curr Drug Saf. 2006;1:1–15.
13. Orton LC, Omari AA. Drugs for treating uncomplicated malaria in pregnant women.Cochrane Database Syst Rev. 2008. CD004912.

14. Chico RM, Hack BB, Newport MJ, Ngulube E, Chandramohan D. On the pathway to better birth outcomes? A systematic review of azithromycin and curable sexually transmitted infections. Expert Rev Anti Infect Ther. 2013;11:1303–32.
15. Kalilani L, Mofolo I, Chaponda M, Rogerson SJ, Alker AP, Kwiek JJ, et al. A randomized controlled pilot trial of azithromycin or artesunate added to sulfadoxine-pyrimethamine as treatment for malaria in pregnant women. PLoS One. 2007;2:e1166.
16. Luntamo M, Kulmala T, Mbewe B, Cheung YB, Maleta K, Ashorn P. Effect of repeated treatment of pregnant women with sulfadoxine-pyrimethamine and azithromycin on preterm delivery in Malawi: a randomized controlled trial. Am J Trop Med Hyg. 2010;83:1212–20.
17. Friedman DS, Curtis CR, Schauer SL, Salvi S, Klapholz H, Treadwell T, et al. Surveillance for transmission and antibiotic adverse events among neonates and adults exposed to a healthcare worker with pertussis. Infect Control Hosp Epidemiol. 2004;25:967–73.
18. Kelsey JJ, Moser LR, Jennings JC, Munger MA. Presence of azithromycin breast milk concentrations: a case report. Am J Obstet Gynecol. 1994;170: 1375–6.
19. Ballard HO, Shook LA, Bernard P, Anstead MI, Kuhn R, Whitehead V, et al. Use of azithromycin for the prevention of bronchopulmonary dysplasia in preterm infants: a randomized, double-blind, placebo controlled trial. Pediatr Pulmonol. 2011;46:111–8.
20. Chang AB, Grimwood K, White AV, Maclennan C, Sloots TP, Sive A, et al. Randomized placebo-controlled trial on azithromycin to reduce the morbidity of bronchiolitis in Indigenous Australian infants: rationale and protocol. Trials. 2011;12:94.
21. Salman S, Rogerson SJ, Kose K, Griffin S, Gomorai S, Baiwog F, et al. Pharmacokinetic properties of azithromycin in pregnancy. Antimicrob Agents Chemother. 2010;54:360–6.
22. Fry AM, Jha HC, Lietman TM, Chaudhary JS, Bhatta RC, Elliott J, et al. Adverse and beneficial secondary effects of mass treatment with azithromycin to eliminate blindness due to trachoma in Nepal. Clin Infect Dis. 2002;35:395–402.
23. Leach AJ, Shelby-James TM, Mayo M, Gratten M, Laming AC, Currie BJ, et al. A prospective study of the impact of community-based azithromycin treatment of trachoma on carriage and resistance of Streptococcus pneumoniae. Clin Infect Dis. 1997;24:356–62.
24. Harding-Esch EM, Edwards T, Mkocha H, Munoz B, Holland MJ, Burr SE, et al. Trachoma prevalence and associated risk factors in the gambia and Tanzania: baseline results of a cluster randomised controlled trial. PLoS Negl Trop Dis. 2010;4:e861.
25. Burr SE, Milne S, Jafali J, Bojang E, Rajasekhar M, Hart J, et al. Mass administration of azithromycin and Streptococcus pneumoniae carriage: cross-sectional surveys in the Gambia. Bull World Health Organ. 2014;92: 490–8.
26. Porco TC, Gebre T, Ayele B, House J, Keenan J, Zhou Z, et al. Effect of mass distribution of azithromycin for trachoma control on overall mortality in Ethiopian children: a randomized trial. JAMA. 2009;302:962–8.
27. Tramper-Stranders GA, van der Ent CK, Gerritsen SA, Fleer A, Kimpen JL, Wolfs TF. Macrolide-resistant Staphylococcus aureus colonization in cystic fibrosis patients: is there transmission to household contacts? J Antimicrob Chemother. 2007;60:665–8.
28. O'Brien KL, Nohynek H. Report from a WHO working group: standard method for detecting upper respiratory carriage of Streptococcus pneumoniae. Pediatr Infect Dis J. 2003;22:133–40.
29. Roca A, Bottomley C, Hill PC, Bojang A, Egere U, Antonio M, et al. Effect of age and vaccination with a pneumococcal conjugate vaccine on the density of pneumococcal nasopharyngeal carriage. Clin Infect Dis. 2012;55:816–24.
30. Brueggemann AB, Pai R, Crook DW, Beall B. Vaccine escape recombinants emerge after pneumococcal vaccination in the United States. PLoS Pathog. 2007;3:e168.
31. Roberts CH, Last A, Molina-Gonzalez S, Cassama E, Butcher R, Nabicassa M, et al. Development and evaluation of a next-generation digital PCR diagnostic assay for ocular Chlamydia trachomatis infections. J Clin Microbiol. 2013;51:2195–203.
32. Bottomley C, Bojang A, Smith PG, Darboe O, Antonio M, Foster-Nyarko E et al.. The impact of childhood vaccines on bacterial carriage in the nasopharynx: a longitudinal study. under review. 2013. Ref Type: Generic
33. Schrag SJ, Verani JR. Intrapartum antibiotic prophylaxis for the prevention of perinatal group B streptococcal disease: experience in the United States and implications for a potential group B streptococcal vaccine. Vaccine. 2013;31 Suppl 4:D20–6.
34. Neal AH, P. Jaon C, Micael AG, Donald SG, Steve K, S. Michael M, et al. Revised guidelines for prevention of early-onset group B streptococcal (GBS) infection. American Academy of Pediatrics Committee on Infectious Diseases and Committee on Fetus and Newborn. Pediatrics.1997;99:489–496.
35. Sigauque B, Roca A, Mandomando I, Morais L, Quinto L, Sacarlal J, et al. Community-acquired bacteremia among children admitted to a rural hospital in Mozambique. Pediatr Infect Dis J. 2009;28:108–13.
36. Onalo R, Ogala WN, Ogunrinde GO, Olayinka AT, Adama SA, Ega BA. Predisposing factors to neonatal septicaemia at ahmadu bello university teaching hospital, zaria Nigeria. Niger Postgrad Med J. 2011;18:20–5.

Submit your next manuscript to BioMed Central and take full advantage of:

- **Convenient online submission**
- **Thorough peer review**
- **No space constraints or color figure charges**
- **Immediate publication on acceptance**
- **Inclusion in PubMed, CAS, Scopus and Google Scholar**
- **Research which is freely available for redistribution**

Submit your manuscript at
www.biomedcentral.com/submit

BioMed Central
