# Supplementary figures and images for: Effect of intra-partum azithromycin on the development of the infant nasopharyngeal microbiota: A post hoc analysis of a double-blind randomized trial
Source: eBioMedicine. 2022 Aug 19;83:104227. doi: 10.1016/j.ebiom.2022.104227 (PMC9420482; doi:10.1016/j.ebiom.2022.104227)

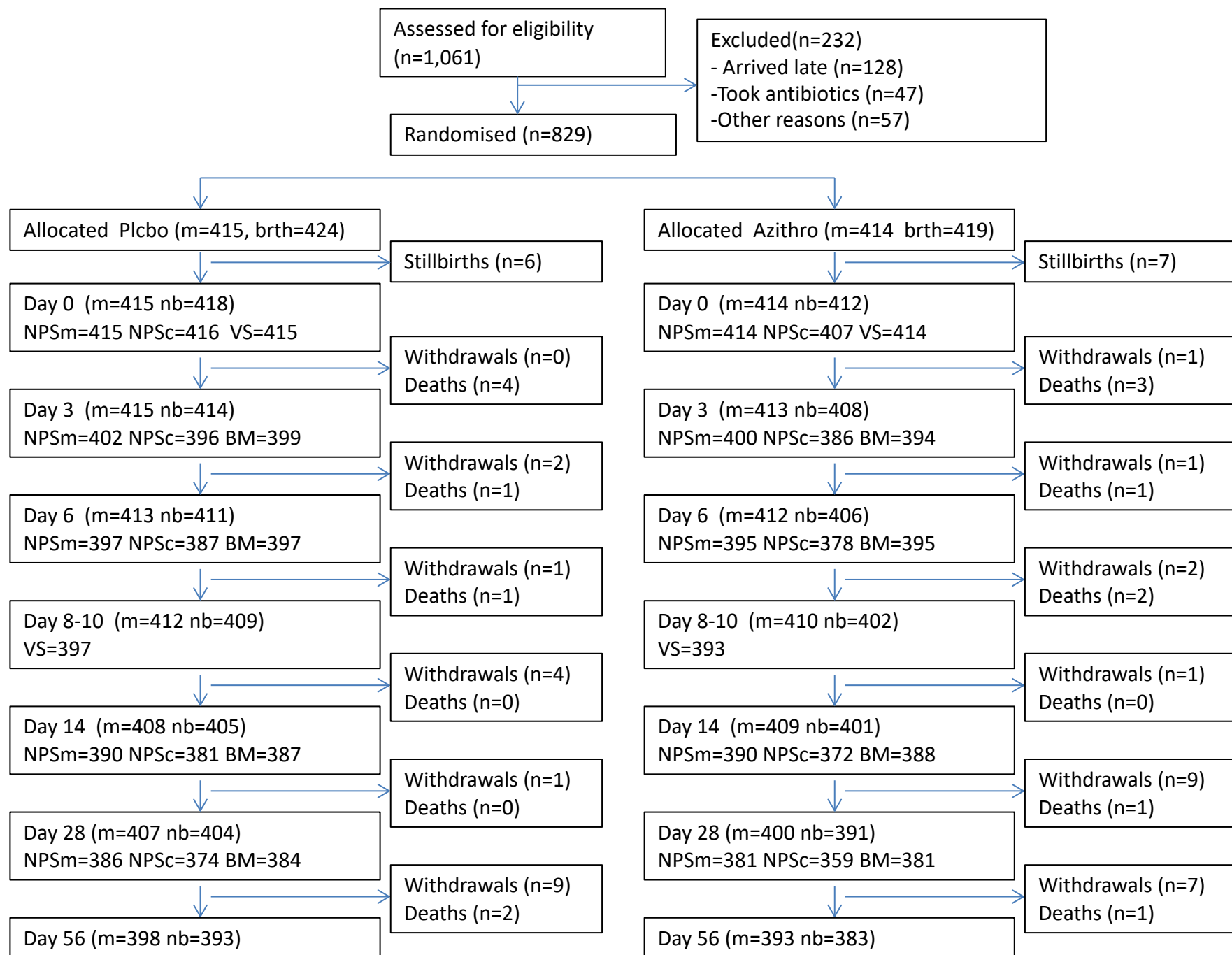

Supplement: Supplementary file 3 [file mmc3.pdf]
